# Species-specific community structure in the microbiomes and eukaryotic communities associated with Mediterranean golf ball sponges

**DOI:** 10.7717/peerj.20452

**Published:** 2026-03-10

**Authors:** Natalie Lewis, Simone Schätzle, Frine Cardone, Dirk Erpenbeck, Gert Wörheide, Sergio Vargas

**Affiliations:** 1 Department of Earth & Environmental Sciences, Palaeontology & Geobiology, LMU München, Munich, Germany; 2 Department of Integrative Marine Ecology, Zoological Station “Anton Dohrn”, Naples, Italy; 3 GeoBio-Center, Ludwig-Maximilians-Universität München, Munich, Germany; 4 SNSB-Bayerische Staatssammlung für Paläontologie und Geologie, Munich, Germany

**Keywords:** Microbiome, Porifera, *Tethya*, Symbiosis, Sponges, Mediterranean

## Abstract

**Background:**

Sponges harbor complex and diverse microbiomes that contribute to the host’s fitness and, ultimately, the health of the ecosystems sponges inhabit.

**Methods:**

Using high-throughput 16S and 18S rRNA amplicon sequencing, we explore the prokaryotic and eukaryotic communities associated with three sympatric Mediterranean demosponges, namely *Tethya aurantium*, *Tethya meloni*, and *Tethya citrina*.

**Results:**

We found species-specific prokaryotic and eukaryotic communities despite the close sympatry of the three Mediterranean *Tethya* species studied. This offers further support for the phylogenetic nature of the sponge microbiome, where microbial communities reflect the evolutionary ancestry of their host species. These patterns are both present in the eukaryotic and prokaryotic sponge-associated communities, since both display similar levels of host species specificity.

## Introduction

The study of the interplay between microbial communities and their hosts has gained significance in both the plant and animal realms (Zilber-Rosenberg & Rosenberg, 2008). These communities collectively form the microbiome (Berg et al., 2020; Whipps, 1991), shaped in part by the host’s environment and its distinctive physicochemical characteristics. While bacterial communities traditionally received primary attention in microbiomes, technological advancements have broadened the concept to encompass eukaryotes, viruses, and fungi (Lecuit & Eloit, 2013; Rodriguez et al., 2009; Vandenkoornhuyse et al., 2015). Within a single microbiome, these groups exhibit significant diversity (Hentschel et al., 2003; O’Dwyer, Kembel & Green, 2012), and microbiomes themselves can vary extensively within specific taxonomic groups. Consequently, microbiomes can be viewed as genomic reservoirs enhancing the host’s biochemical and physiological capabilities (Dubilier, Bergin & Lott, 2008; Nicholson et al., 2012; Wilson et al., 2010).

Several studies (Kennedy et al., 2014; Moitinho-Silva et al., 2014; Reveillaud et al., 2014) indicate the stability of sponge microbiomes, distinguishing them from microbial communities in the surrounding water. These microbiomes exhibit both vertical and horizontal microbial transmission (Reveillaud et al., 2014; Schmitt et al., 2012; Taylor et al., 2013; Webster et al., 2010), with the significance of these symbiont acquisition forms varying across sponge groups and members of the sponge microbiome (Carrier et al., 2022). Despite the occurrence of horizontal symbiont acquisition, sponge microbiomes still display a phylogenetic signal, with higher microbiome similarity observed within phylogenetically close groups (Benson et al., 2010; Doane et al., 2020; Lim & Bordenstein, 2020; O’Brien et al., 2020). However, most studies have predominantly focused on the prokaryotic component of the sponge microbiome, neglecting the substantial numbers of sponge-associated eukaryotes (Hardoim et al., 2021). A comprehensive characterization of all taxonomic groups associated with a specific sponge species is essential for understanding the sponge holobiont and its responses to environmental changes triggered by climate change (Pineda et al., 2017; Thomas et al., 2016; Vargas, Leiva & Wörheide, 2021). Additionally, such characterization facilitates cross-taxonomic comparisons to assess whether similar ecological and evolutionary processes shape the diversity of different components of the sponge holobiont (Busch et al., 2022; Hentschel, Usher & Taylor, 2006; Thomas et al., 2016).

In this study, we employed high-throughput sequencing of the V4 16S rRNA and the V9 18S rRNA to investigate the diversity and composition of prokaryotic and eukaryotic communities associated with three sympatric Mediterranean *Tethya* species: *T. aurantium* (Pallas et al., 1766), *T. citrina* Sarà & Melone, 1965, and *T. meloni* (Corriero, Gadaleta & Bavestrello, 2015). These *Tethya* species coexist in open and transitional environments with overlapping occurrences, spanning a diverse range of Mediterranean biotopes, habitats, and substrates (Sarà & Melone, 1965; Pansini & Pronzato, 1973; Corriero, Balduzz & Sarà, 1989). Despite extensive ecological and reproductive studies on these species (Connes, 1971; Liaci et al., 1971; Corriero, Sarà & Vaccaro, 1996; Corriero, Balduzz & Sarà, 1989; Gaino et al., 2006; Cardone, Corriero & Gaino, 2010), their microbiomes, particularly their eukaryotic associates, remain poorly characterized. The close co-occurrence of these species in certain Mediterranean localities provides a unique opportunity to investigate how phylogenetic and ecological processes influence the composition of prokaryotic and eukaryotic communities associated with closely related sponge species occurring in sympatry. Our findings underscore the presence of species-specific microbiome in sympatric Mediterranean *Tethya*, suggesting that similar processes shape the composition of sponge-associated eukaryotic communities.

## Materials & Methods

### Sample collection

**Figure 1:**
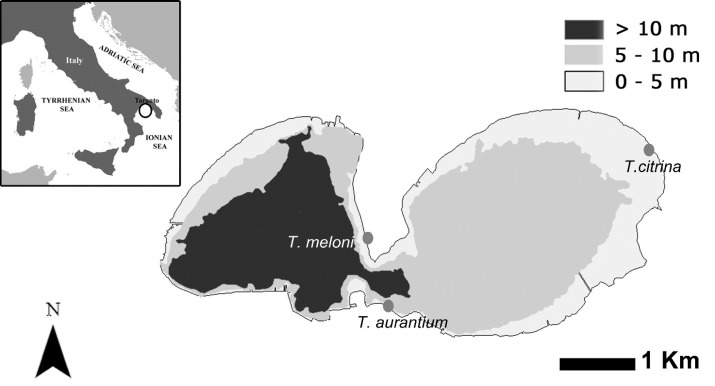
Map of sampling sites for *T. aurantium.*, *T. meloni* and *T. citrina* at Mar Piccolo. Colour indicates depth of the lagoon and the grey dots represent the sampling location for each species.

All the samples were collected in Mar Piccolo (40°30′07.17″N–17°15′47.61″E), a small sea located at the northern end of the Gulf of Taranto (Ionian Sea, Central Mediterranean). Mar Piccolo is an inner, semi-enclosed basin with a total surface area of approximately 20.63 km^2^ (Fig. 1). This unique area displays lagoon-like features and is divided by two rocky promontories into two distinct inlets called the Primo Seno and Secondo Seno. The Primo Seno has a maximum depth of 13 m, while the Secondo Seno reaches a maximum depth of eight m.

The three *Tethya* species investigated here exhibit a patchy distribution with specific habitat preferences in the Mar Piccolo basin. *T. citrina* is exclusively found in the Secondo Seno, generally on the surfaces of anthropogenic stones and boulders shielded from direct light, and up to approximately 1 meter depth. It can reach densities of approximately 100 individuals per square meter of substrate (Cardone et al., 2010). Conversely, *T. meloni* is primarily distributed in the Primo Seno, where it predominantly colonizes artificial substrates such as mussel farming poles and artificial jetties, at depths ranging from one to five meters. In certain areas, this species shows a strong inclination to form persistent assemblages consisting of large individuals (approximately seven cm in diameter) at high densities (>50–100 individuals per square meter). Lastly, *T. aurantium* is also primarily found in the Primo Seno, at depths ranging from 0.5 to 2 m, where it inhabits the walls of small rocky crevices or rocks on the seafloor. A total of 44 *Tethya* samples were collected in this study. These samples consisted of 12 specimens of *T. aurantium*, 13 specimens of *T. meloni*, and 19 specimens of *T. citrina*. To collect the samples, sponge pieces were carefully cut while ensuring the preservation of the specimens’ integrity. Following collection, the sponge pieces were immediately fixed in absolute ethanol for further analysis.

### Extraction and high throughput sequencing and amplification of 16S rRNA and 18S rDNA

DNA was extracted using the Macherey-Nagel NucleoSpin DNA extraction kit following the manufacturer’s protocol, quantified using a Nanodrop 1000, quality controlled using 1% agarose gels, and diluted to a concentration of five ng/µl before amplification. The V4 region of the 16s rRNA was amplified from each sample using barcoded forward and reverse primers 515f and 806rB, respectively, following Pichler et al. (2018). To characterize the eukaryotic community, we adapted the method developed by Pichler et al. (2018) to amplify the V9 region of the 18S rRNA using barcoded primers Euk1391f and EukBr (Table S1). PCR products were visualized on 1.5% agarose gels, and bands of the expected size (*i.e.,* ∼380 bp for 16S rRNA and ∼270 bp for 18S rRNA) were cut out under UV light and purified using Macherey-Nagel NucleoSpin Gel and PCR Cleanup kit. After purification, DNA concentration was determined using a Qubit 3.0 fluorometer (Life Technologies, Grand Island, NY, USA), and the samples were diluted to 2 nM or 1nM before equimolar pooling. The 16S and 18S rRNA amplicon pools were sequenced on an Illumina MiniSeq in 300PE mode following Pichler et al. (2018).

### Sequence processing and OTU classification

The obtained Illumina 16S and 18S rRNA sequence reads were demultiplexed to their respective samples according to their unique barcode combinations and processed independently using a standardized pipeline in the program *vsearch* (Rognes et al., 2016). The pipeline was used to combine the two reads into contigs, filter chimeras and singletons, cluster sequences into operational taxonomic units (OTUs) of 97% similarity, and count the number of reads obtained for each OTU. After running *vsearch*, the OTU count matrix and the OTU centroid sequences were used in further analyses. The taxonomic affinity of the resulting OTUs was determined using the Silva database (Quast et al., 2013) with a 95% minimum identity between the query (*i.e.,* the OTU centroids) and the SILVA reference sequence. The taxonomic ranks were downloaded and manually adjusted to include: Domain, Phylum, Class, Order, Family, Genus, and Species. Other ranks found in the 18S rRNA dataset, such as Subclass, group, and supergroup, were excluded from the analysis.

### Community analyses

The taxonomic classification and OTU count matrices were combined in R version 4.0.2 (R Core Team, 2020) using the OTU identifiers as merging keys. For the 16S rRNA data, OTUs with less than fifty counts were excluded from further analyses. For the 18S rRNA data, since the eukaryotic communities had lower read counts allocated to the different OTUs, the threshold for OTU exclusion was lowered to five counts instead of 50. Also, to account for sponge 18S rRNA co-amplification, the two most abundant OTUs were removed from all subsequent analyses; these OTUs were consistently assigned to Demospongiae by Silva. Unexpectedly, bacterial OTUs were also observed in the 18S rRNA data, and *in silico* tests revealed that primer Euk1391f and EukBr bind the 3′region of the bacterial 16S rRNA. Thus, these OTUs were also removed from the eukaryotic dataset. For the 18S and 16S rRNA datasets, the package *vegan* 2.5-7 (Oksanen et al., 2020) was used in R to estimate rarefaction curves as a proxy for sampling completeness (Figs. S1 and S2).

For each *Tethya* species, the prokaryotic and eukaryotic phylum-level richness and the core community were identified. The core community was defined as those OTUs present in at least 90% of the samples available for a species. Beta diversity was analyzed using Bray–Curtis dissimilarities and non-metric multidimensional scaling (NMDS) ordination plots to investigate compositional differences between the prokaryotic and eukaryotic communities of the *Tethya* species. To identify whether microbial community composition relates to host species identity Canonical Correspondence Analyses implemented in the vegan package (anova, 999 permutations) were used. Microbial community dissimilarities, displayed in NMDS plots, were calculated using the Jaccard index and their significance assessed with permanova tests (adonis2, 999 permutations). Finally, a multivariate dispersion test (vegan; betadisper) was used to compare variability within species and to test assumptions of the permanova based on homogeneity of group variability. Tests were run separately for the prokaryotic and eukaryotic data for all three species; *post hoc* pairwise analyses were done to compare species pairs.

To visualize the most variable OTUs on a heatmap, the variance of each OTU was calculated across all samples using the matrixStats package in R. OTUs were ranked according to their variance, and the 25 taxa with the highest variability were selected for representation in a heatmap. Prior to plotting, abundances were standardized by *z*-score transformation (subtracting the mean and dividing by the standard deviation for each OTU), ensuring comparability across taxa with different overall abundance levels.

Also, OTU rank-abundance dominance (RAD) curves were estimated for each sample. Several RAD models were fit to the OTU data, and the best-fit RAD model was determined using Akaike’s information criterion (AIC) in vegan (v2.5-7) (Oksanen et al., 2020). Samples were excluded from the RAD analyses if it was not possible to fit RAD models due to lack of convergence or the rarefaction curves suggested undersampling (*i.e.,* sample GW1956 for the 16S rRNA dataset and sample GW1968 for the 18S rRNA dataset; see Figs. S1 and S2).

## Results

### Phylum- and OTU-level richness of the prokaryotic community associated with Mediterranean *Tethya*

**Figure 2:**
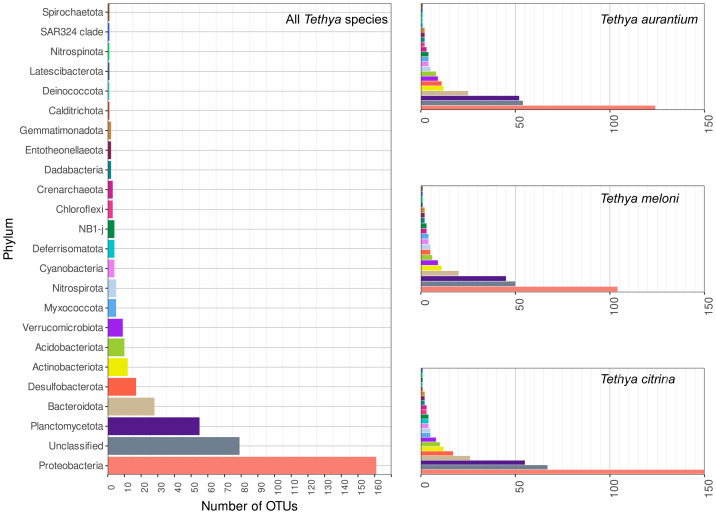
16S OTUs richness across phyla present for all species (left) and the three *Tethya* species separately (right). The color of the bars in all plots is indicative of the taxonomic group.

We identified 4,075 prokaryotic OTUs from the generated 16S rRNA sequences. Of these, only 411 surpassed the set coverage threshold (≥50 counts). These OTUs were distributed across 23 phyla, with 79 OTUs remaining unclassified. The richest phylum observed was Proteobacteria, with 161 OTUs, followed by Planctomycetota (55 OTUs), Bacteroidota (28 OTUs), Desulfobacterota (17 OTUs), Actinobacteria (12 OTUs), Verrumicrobiota (nine OTUs), and ten other phyla represented by five or fewer OTUs (Fig. 2). These phyla were consistently present in all three *Tethya* species. Some phyla, such as Deferrisomata (four OTUs), Chloroflexi (three OTUs), Calditrichota (one OTU), and Deinococcota (one OTU), were detected in *T. aurantium* and *T. citrina* but were absent in all *T. meloni* samples. Calditrichota and Latescibacterota (one OTU) were not found in *T. aurantium*, and Spirochaetota (one OTU) was absent in *T. citrina*.

**Figure 3:**
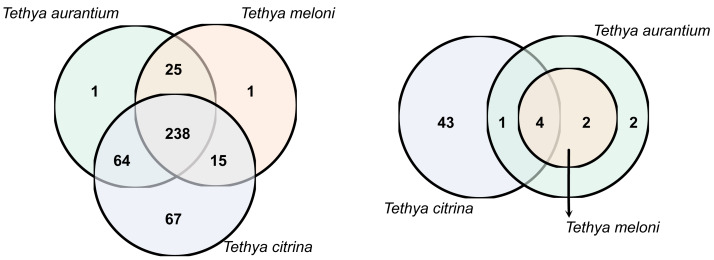
16S OTU overlap between species of Mediterranean *Tethya.* Left: whole microbiome. Right: core 16S OTUs (*i.e.,* OTUs present in ≥90% of a species’ samples).

*Tethya citrina* exhibited the highest number of OTUs (384), followed by *T. aurantium* (328) and *T. meloni* (279). Over half of the OTUs (58%) were shared among all three species, while 25% were shared between two *Tethya* species (Fig. 3). The remaining 17% consisted of OTUs specific to one *Tethya* species, with 97% belonging to *T. citrina*. Consequently, the percentage of OTUs exclusively found in *T. aurantium* is approximately 0.3%, in *T. meloni* is 0.36%, and in *T. citrina*, 17%.

**Figure 4:**
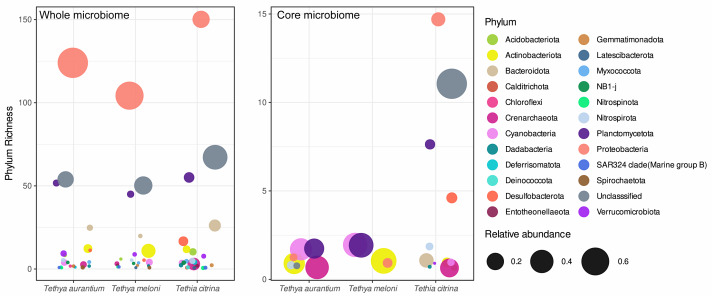
Phylum OTU richness and relative abundance in the whole and core microbiome of Mediterranean *Tethya* species.

Examining the core communities of the three *Tethya* species, *T. citrina*’s core community was the most OTU-rich (Fig. 3), with 43 exclusive OTUs spanning various phyla, including Proteobacteria (14), Desulfobacterota (5), Nitrospirota (2), Verrucomicrobiota (1), Acidobacterora (1), Dadabacteria (1), and Bacteroidota (1); 11 OTUs remained Unclassified by the SILVA database (Fig. 4). In contrast, *T. aurantium* maintained two exclusive core OTUs: the unclassified OTU 54 and Nitrospira sp. (OTU 124), while *T. meloni* had no exclusive core OTUs. Four core OTUs were shared across all investigated *Tethya* species, including OTU 21 of the PeM15 Order (Actinobacteria), OTU 25 Synechococcus CC9902 (Cyanobacteria), OTU 52 of the Pirellulaceae family (Planctomycetota), and OTU 43 of the SAR11 clade Ia (*α*-Proteobacteria). *Tethya aurantium* and *T. meloni* shared two OTUs: OTU 27, also belonging to the Pirellulaceae, and OTU 65, identified as Cyanobium PCC-6307 (Cyanobacteria). Additionally, *T. aurantium* shared one core OTU, the archaeon Candidatus Nitrosopumilus (OTU 11), with *T. citrina*. No core OTUs were shared exclusively between *T. meloni* and *T. citrina*.

### Phylum and OTU-level relative abundance

Proteobacteria emerges as the dominant phylum in *T. aurantium* and *T. meloni*, closely followed by unassigned OTUs and the phylum Actinobacteria (Fig. 4). In contrast, *T. citrina* exhibits a higher proportion of OTUs with unknown phylum affiliation and a significantly lower abundance of Proteobacteria relative to the other species. Notably, the phyla Bacteroidota, Crenarchaeota, Desulfobacterota, and Planctomycetota are notably abundant in *T. citrina* (Fig. 4).

At the OTU level, proteobacterial OTU 1 (a gamma-proteobacterium in the EC94 family) and OTU 2 (an alpha-proteobacterium in the Rhodobacteraceae family) dominate in *T. aurantium* and *T. citrina* samples. Interestingly, these two OTUs appear in *T. citrina* at lower relative abundances (Fig. S3). *T. citrina* exclusively harbors another abundant proteobacterial OTU, OTU 10 (Family Rhodobacteraceae), albeit at a significantly lower average relative abundance per sample than other proteobacterial OTUs in *T. aurantium* and *T. meloni*. Conversely, the archaeal OTU 11 (Candidatus Nitrosopumilus) is the most abundant OTU on average in *T. citrina.* This OTU occurs at lower abundances and in fewer samples in *T. aurantium* and *T. meloni*.

In *T. citrina*, four unclassified OTUs, absent in other *Tethya* species, and a Bacteroidetes OTU belonging to the genus *Ekhidna* occupy the next abundance ranks. Interestingly, unclassified OTUs that are more abundant in the other two *Tethya* species are not prominent in *T. citrina*. A representative of Actinobacteria (OTU 11; PeM15) exhibits abundance across all *Tethya* species. Additionally, three Actinobacteria OTUs assigned to the Sva0996 in the Michotrichaceae are abundant in *T. citrina*, with two of these OTUs also displaying abundance in *T. meloni*. Notably, the relative abundance of OTUs is more consistent across samples of *T. citrina* compared to *T. aurantium* and *T. meloni* (Fig. 4; Figs. S3 and S4).

### Rank-abundance dominance models

**Figure 5:**
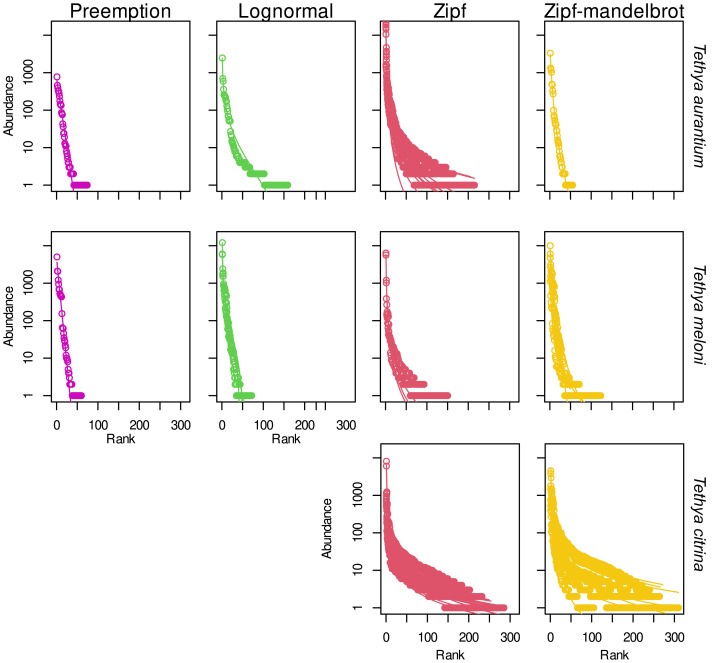
Abundance-rank dominance models fitted to OTU data for the 16S dataset. For each species (A) *T. aurantium,* (B) *T. meloni,* (C) *T. citrina*), the sample replicates (represented as lines) are drawn under the best-fit model for the distribution of the OTU count data (represented as circles) and grouped into the same box under the name of the assigned model.

The Zipf model emerged as the best-fit model based on AIC for 57.1% of samples, with the Zipf-Mandelbrot model closely following as the preferred fit for 28.6% of the samples. The lognormal and preemption models were identified as the best fit for 9.5% and 4.8% of the samples, respectively (Fig. 5). In the case of *T. aurantium*, the Zipf model best fit nine samples, while the niche preemption, Zipf-Mandelbrot, and lognormal models were the optimal fit for one sample each. For *T. meloni*, the Zipf-Mandelbrot, Zipf, and lognormal models were chosen as the best-fit models for three samples each. The niche preemption model was identified as the best-fit model for only one sample, and one sample failed to converge in this species. As for *T. citrina*, only the Zipf and Zipf-Mandelbrot models were selected as the best fit models for twelve and seven samples, respectively. Despite the prevalence of the Zipf model as the best fit for most samples, the difference in deviance between the models is not significant for any of the three *Tethya* species (Fig. S5).

### *Tethya* species-specific community structure

**Figure 6:**
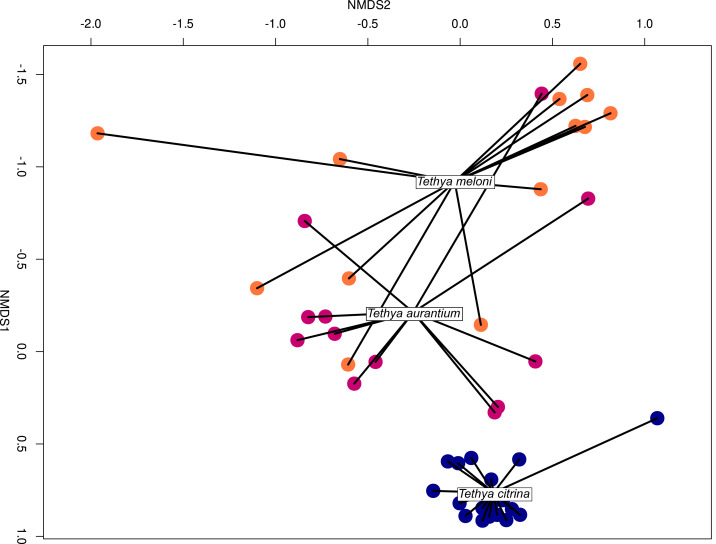
NMDS ordination plots of Bray-Curtis 16S community dissimilarities across samples Mediterranean samples of *T. aurantium*, *T. meloni*, and *T. citrina*. Each dot represents one sample. *Tethya meloni* samples are peach-colored, *T. aurantium* samples are colored purple-red, and *T. citrina* samples are blue.

Host identity for all three *Tethya* species significantly explained prokaryotic microbial community composition (*F* = 9.32, *df* = 2, *p* = 0.001; permutation test with 999 permutations; Table S2). Despite the apparent community overlap between *T. meloni* and *T. aurantium*, these two species significantly differ in the *post-hoc* pairwise correspondence analysis (*F* = 7.19, *df* = 1, *p* = 0.006; permutation test with 999 permutations). Furthermore, NMDS analyses utilizing only presence-absence data derived from the community matrix also support the observed microbiome differentiation in the analyzed *Tethya* species (Fig. 6; PERMANOVA, *F* = 10.5, *R*^2^ = 0.34, *p* = 0.001), with host species identity explaining ∼34% of the variation. However, tests of homogeneity of multivariate dispersion revealed highly significant differences in within-group variability (beta dispersion: *F*_(2,41)_ = 17.25, *p* = 3.68 × 10^−6^; permutation test: *p* = 0.001). Tukey’s HSD tests showed that *T. citrina* exhibited significantly lower dispersion than both *T. aurantium* (*p* = 0.0014) and *T. meloni* (*p* = 4.4 × 10^−6^), whereas *T. aurauntium* and *T. meloni* did not differ significantly (*p* = 0.27). These results indicate that although species identity significantly structures microbial communities (Fig. S6), differences in group variability (dispersion) also contribute to the overall separation observed. An abundance-based cluster analysis of the 25 most variable OTUs further underscores some species-specific structure (Fig. S7), with samples of the same species grouping into approximately three clusters per species. Similar analyses considering all OTUs highlight the distinct separation of *T. citrina* from other *Tethya* species. However, *T. meloni* and *T. aurantium* become more challenging to distinguish in those analyses (Fig. S8).

### Phylum and OTU-level richness of the *Tethya*-associated eukaryotic community

**Figure 7:**
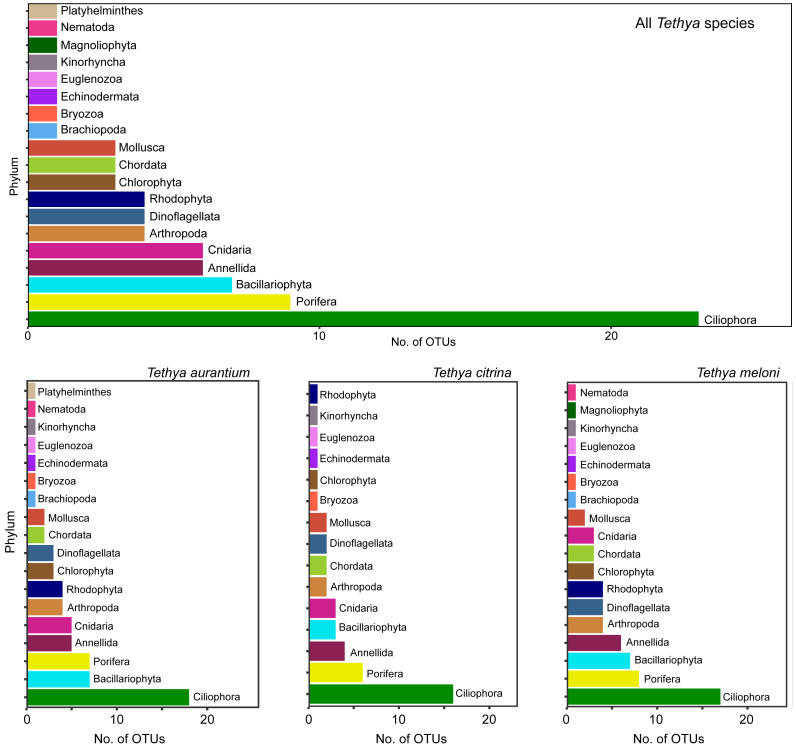
18S OTUs richness across phyla present for all species (top) and the three *Tethya* species separately (bottom). The color of the bars in all plots is indicative of the taxonomic group.

In the 18S dataset, a total of 1,018 OTUs were initially identified. However, among these OTUs, 189 were bacterial, and several had coverages below the set threshold value of five read counts for the 18S rRNA dataset (see Materials and Methods). After excluding bacterial OTUs, low-coverage OTUs, and the two most abundant OTUs representing co-amplified sponge DNA, only 295 OTUs were retained for further analysis. *Tethya aurantium* exhibited the highest OTU richness (227 OTUs), followed by *T. meloni* (153 OTUs) and *T. citrina* (136 OTUs) (Fig. 7). Of these OTUs, 215 remained unclassified. Among the classified OTUs, 19 eukaryotic phyla were identified, including four microeukaryotes: Ciliophora (23 OTUs), Euglenozoa (one OTU), Bacillariophyta (seven OTUs), and Dinoflagellata (four OTUs), two phyla representing either macroalgae or marcrosopic photosynthetic eukaryotes: Rhodophyta (four OTUs) and Chlorophyta (three OTUs), and nine macroeukaryotic phyla: Porifera (nine OTUs), Annelida (six OTUs), Cnidaria (six OTUs), Arthropoda (four OTUs), Chordata (three OTUs), Mollusca (three OTUs), Bryozoa (one OTU), Echinodermata (one OTU), and Kinorhyncha (one OTU) (Fig. 7). These phyla were present in all three *Tethya* species analyzed. Nematoda (one OTU) and Brachiopoda (one OTU) were absent in any *T. citrina* samples, and Platyhelminthes (one OTU) was only detected in *T. aurantium*. Magnoliophyta (one OTU) was exclusively found in *T. meloni*. 29 classes were identified within the identified phyla.

**Figure 8:**
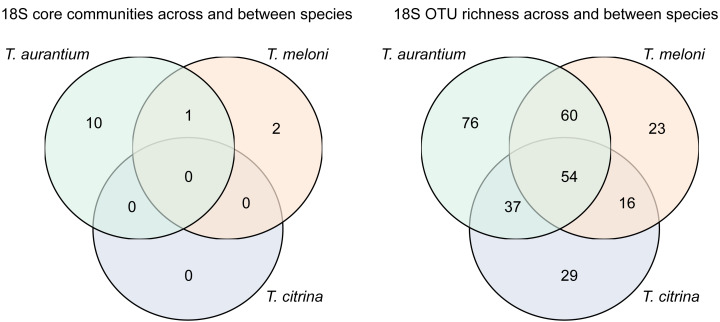
18S OTU overlap between species of Mediterranean *Tethya.* Left: core community (OTUs present in 90% of samples). Right: all 18S OTUs.

For all three *Tethya* species, Ciliophora emerged as the most species-rich eukaryotic phylum. Porifera, Bacillariophyta, and Annelida followed in OTU richness, with some variability in their order (Fig. 7). Despite the substantial similarity in phylum-level richness, over half of the detected OTUs were present at least once in more than one *Tethya* species. However, many OTUs were exclusively detected in one of the *Tethya* species, particularly in *T. aurantium* (Fig. 8). In this context, the eukaryotic OTUs demonstrated less similarity between *Tethya* species than prokaryotic OTUs (as mentioned above). About 18% of all OTUs were present in all three *Tethya* species, and 38% were in two of the three *Tethya* species. The number of species-specific eukaryotic OTUs was 33% in *T. aurantium*, 21% in *T. citrina*, and 15% in *T. meloni*. Unlike the prokaryotic community, a eukaryotic core community could not be defined for *T. citrina*, while the eukaryotic core communities of *T. aurantium* and *T. meloni* mainly comprised unclassified OTUs.

### Eukaryotic phylum- and OTU-level relative abundance in Mediterranean *Tethya*

**Figure 9:**
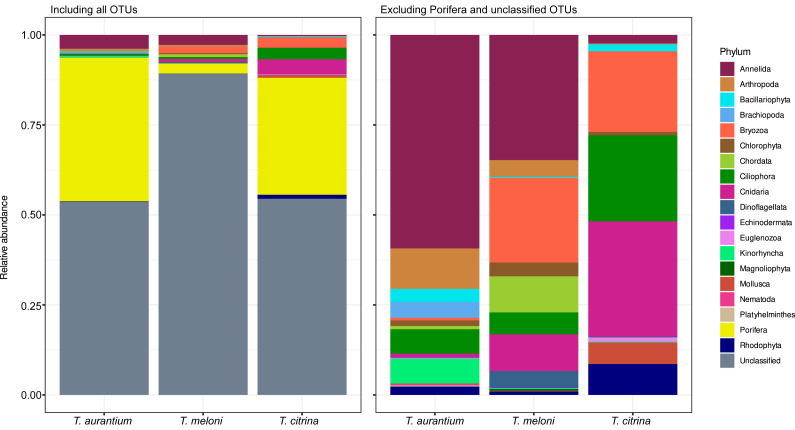
Relative abundance of 18S OTUs grouped into phyla for each *Tethya* species for (A) all OTUs and (B) excluding unclassified OTUs and OTUs belonging to Phylum Porifera.

Concerning abundance, unclassified eukaryotic OTUs dominate the community in all three *Tethya* species, followed by OTUs assigned to the phylum Porifera (Fig. 9). Compared to Porifera, the disparity in abundance of OTUs belonging to unclassified phyla is less pronounced in *T. aurantium* and *T. citrina*, but significantly greater in *T. meloni*, where unclassified OTUs dominate. However, with only the unclassified OTUs removed, *T. meloni* exhibits a lower abundance of Porifera OTUs than Annelida and shares abundance more evenly across the remaining phyla. Excluding Porifera OTUs, *T. aurantium* appears to be the most diverse, while *T. meloni* and *T. citrina* demonstrate greater evenness (Fig. 9). In *T. aurantium*, Annelida is relatively abundant, and in *T. citrina*, Nematoda becomes the most abundant phylum, followed by Ciliophora and Bryozoa.

The high relative abundance of Annelida in *T. aurantium* likely results from the high abundance in three samples of Phyllodocida OTU 4087 (Fig. S9). OTU 4090, belonging to the order Phyllodocida, is only present at high abundance in a single sample of *T. meloni*, explaining the elevated Annelida abundance in that species. However, OTU 2087 is also present in *T. meloni* at low abundance, and both Phyllodocida occur in *T. citrina* at low abundance. Bryozoa is represented by a single OTU, 4101 of the order Ctenostomatida, highly abundant in two *T. meloni* and two *T. citrina* samples. The elevated relative abundance of Cnidaria in *T. citrina* primarily results from OTU 4146, a hydrozoan in the Leptothecata order present in two samples, and OTU 4212, an anthozoan in Actiniaria present in three samples.

In contrast, despite its higher OTU richness, Ciliophora has only a single OTU in a single *T. citrina* sample, occurring at approximately 0.06 abundance (OTU 4286 of Class Oligohymenophorea). Therefore, the high relative phylum-level abundance must result from the combination of several lower-abundance Ciliophora OTUs (Fig. S9). Rhodophyta and Bacillariophyta also display this abundance pattern.

### Rank-Abundance Dominance (RAD) models for the eukaryotic community

**Figure 10:**
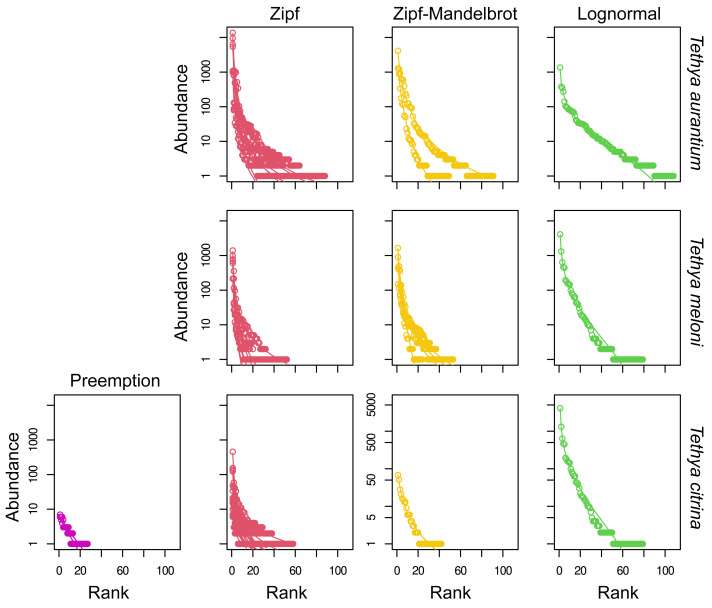
Rank-abundance dominance models fitted to OTU data for the 18S dataset. For each species (*i.e., T. aurantium, T. meloni,* and *T. citrina*), the replicates (represented as lines) are drawn under the best-fit model for the distribution of the OTU count data (represented as circles) and grouped into the same box under the name of the assigned model.

The Zipf model emerged as the best-fit RAD model for the eukaryotic community, with the lowest AIC in 70% of samples. The Zipf-Mandelbrot model was the optimal fit for 17.5% of samples, while the lognormal and preemption models were the best fit for 7.5% and 5% of the samples, respectively (Fig. 10). Among *T. aurantium* samples, the Zipf model best fit seven samples, the Zipf-Mandelbrot model two samples, and the lognormal model one sample. Two samples were excluded as they did not converge. For *T. meloni*, the Zipf model was the best fit for seven samples, followed by the Zipf-Mandelbrot model for four samples, and the lognormal model for one sample. An additional sample was excluded due to convergence issues. In the case of *T. citrina*, the Zipf model was the best fit for fourteen samples, the preemption model for two samples, and the Zipf-Mandelbrot and lognormal models for one sample each. Similar to the prokaryotic dataset, although the Zipf model prevailed as the best fit for most samples, the difference in deviance between the models was not significant for any of the three *Tethya* species (Fig. S10). Additionally, the Zipf model did not exhibit a significant advantage over the Zipf-Mandelbrot or the lognormal model.

### *Tethya* eukaryotic microbiomes show a species-specific community structure

**Figure 11:**
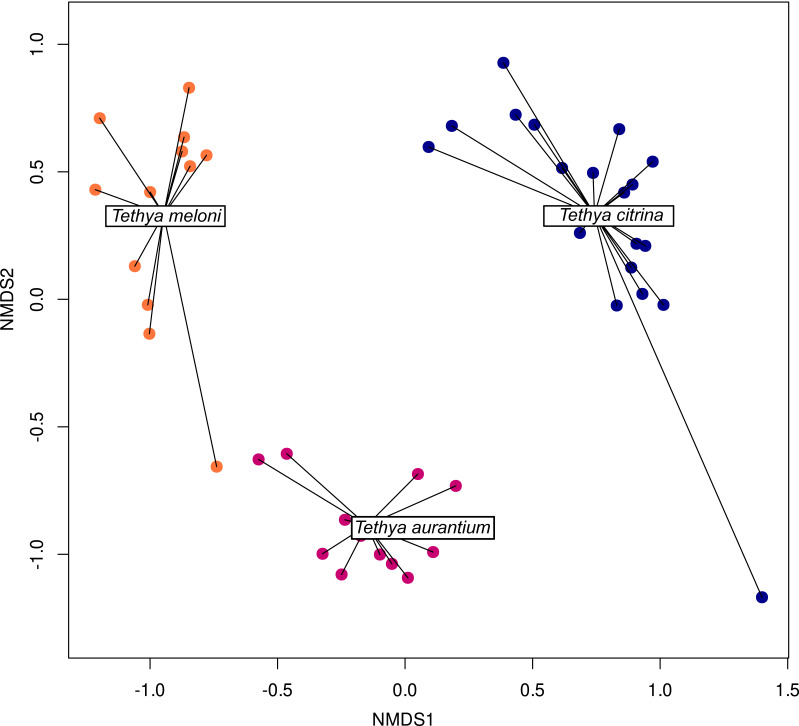
NMDS ordination plots of Bray-Curtis distances for 18S community structure across samples grouped by species. Each dot represents one sample. *Tethya meloni* samples are peach-colored, *T. aurantium* samples are colored purple-red, and *T. citrina* samples are blue.

The CCA shows a species-specific eukaryotic structure both for all three species (*F* = 4.26, *df* = 2, *p* = 0.001) and for the two more similar *T. meloni* and *T. aurantium* (*F* = 3.39, *df* = 1, *p* = 0.001). The Non-metric Multidimensional Scaling analysis (NMDS ordination plot; Fig. 11) reveals a distinct species-specific eukaryotic community structure in Mediterranean *Tethya* (PERMANOVA, *F* = 7.06, *R*^2^ = 0.26, *p* = 0.001). Individuals of the same species exhibit eukaryotic communities with greater similarity within species than between species. Importantly, these patterns are not attributed to changes in abundance, as evidenced by the clear differences observed in the presence-absence analyses of the eukaryotic microbiomes studied (Fig. S11). However, as with the prokaryotic microbial communities, tests of homogeneity of multivariate dispersion revealed highly significant differences in within-group variability (beta dispersion: F(2,41) = 5.1, *p* = 0.011; permutation test: *p* = 0.011).

Clustering based on the 25 most variable OTUs in abundance also demonstrates a general species-specific pattern (Fig. S12), although some specimens deviate from this overall trend.

## Discussion

In our investigation of the prokaryotic and eukaryotic associates of the Mediterranean sponge species *T. aurantium*, *T. citrina*, and *T. meloni*, we observed bacterial communities within the established sponge range, aligning with quantities in a previously described *Tethya* species (Thomas et al., 2016). Phyla composition matched prior sponge microbiome studies, including Proteobacteria, Planctomycetota, Bacteroidota, and others (Schmitt et al., 2012; Taylor et al., 2013; Thomas et al., 2016; Santodomingo et al., 2024). Phyla like SAR324 clade, Entotheonellaeota, NB1-j, Myxococcota, Nitrospirota, Desulfobacterota, and Deferrisomatota, previously classified under Deltaproteobacteria, were identified in our study (Engelberts et al., 2020; Waite et al., 2020; Robbins et al., 2021). Consistency in bacterial phyla composition across both samples and studies supports the conclusion that even in low microbial abundance sponges, microbial communities could be used as an alternative approach for species identification (Sipkema & Blanch, 2010; Santodomingo et al., 2024)

OTU-richness patterns resembled those in a previous study (Thiel et al., 2007). Shared OTU richness among *Tethya* species suggests evolutionary and environmental influences. This trend extends to the core community, where *T. aurantium* and *T. meloni* share primarily generalist core OTUs, while *T. citrina* exhibits a more specialized core, including Pirellulaceae implicated in nitrogen cycling.

Phylum relative abundance varied between species, with *T. citrina* displaying a more even microbiome despite higher Proteobacteria richness. The relatively low evenness in *T. aurantium* and *T. meloni* was attributed to non-core Proteobacteria dominance in some samples. Contrary to typical LMA sponge microbiomes, *T. citrina* did not exhibit dominance by a single Proteobacteria or Cyanobacteria taxon. Despite differences, most OTUs occurred at low relative abundances across all three species. Abundance-rank models, Zipf and Zipf-Mandelbrot, revealed a transition to higher evenness in the rare fraction of the community, capturing this pattern.

Mediterranean *Tethya* species exhibit a species-specific eukaryotic community, consistent with previous research on sponge-associated eukaryotes (Hardoim et al., 2021). Our study identified 13 microbial eukaryotic phyla, slightly fewer than the 15 reported by Hardoim et al. (2021), with 73% of 18S OTUs remaining unclassified. Notably, aside from Porifera, Kinorhyncha, and Magnoliophyta, all detected phyla have been previously described in sponges (Chaib De Mares et al., 2017). Kinorhyncha, small free-living worms associated with sponges, were consistently found in all specimens.

Despite Poriferan OTUs likely resulting from co-amplification, our study identified OTUs classified as Calcareous and Clionaid sponges among high-abundance eukaryotic 18S OTUs in Mediterranean *Tethya*. This suggests the possible detection of other sponge species within the *Tethya* eukaryotic community. At the Class level, our investigation identified 29 eukaryotic classes, slightly fewer than the 32 reported in a previous study, excluding macro-eukaryotes (Hardoim et al., 2021).

The shared richness in the eukaryotic community was lower than in the prokaryotic community, with 18% of 18S OTUs present in all species and 57% in at least two *Tethya* species. Species-specific 18S OTUs ranged from one-third in *T. aurantium* to 15% in *T. meloni*. Unlike studies focusing on phylogenetically diverse sponges, which reported over 50% to 82.6% of 18S OTUs as species-specific, our findings reveal no phylosymbiotic signal in eukaryotic communities between closely related sponge species (Chaib De Mares et al., 2017; He et al., 2014), despite the strong species-specificity observed in these communities.

A potential limitation of our study was the use of 18S primers capable of amplifying sponges, resulting in a dominance of Porifera and unclassified OTUs. Improved primers or blocking oligonucleotides could enhance the identification of *Tethya*-associated eukaryotes. However, the decision to exclude Porifera from the 18S pool should be approached carefully, as it may exclude sequences from other sponge species near the sampled specimens or from larvae settling on or trapped in the *Tethya* species sampled. The large number of unclassified OTUs suggests further investigation and classification of microbial diversity are needed, but since OTUs were not cross-referenced with GenBank, sequencing errors cannot be fully excluded. Despite this, we expect unidentified OTUs to be more indicative of unexplored biodiversity than sequencing errors since chimeric sequences were excluded in the pipeline. One interesting result of the present study is that community structure is species-specific, with greater similarity within species samples than between species for both the prokaryotic and eukaryotic communities. *Tethya citrina* had the richest prokaryotic community, and *T. aurantium* the richest eukaryotic community. However, regarding diversity (evenness), the most diverse *Tethya* species for overall prokaryotic and eukaryotic communities is *T. citrina*. This distinction could be due to the similarity between *T. aurantium* and *T. meloni*. These species are of similar size and have a well-developed cortex that contrasts with the cortex of *T. citrina*, which is thinner (Corriero et al., 2007).

Interestingly, a denser mesophyll, which reduces water flow, has long been linked to HMA sponges (Gloeckner et al., 2014; Schläppy et al., 2010; Vacelet & Donadey, 1977; Weisz, Lindquist & Martens, 2008); however, our results partly contradict this, as *T. aurantium* and *T. meloni* had lower microbial diversity than *T. citrina*. Our results further support recent findings pointing to host-specific eukaryotic microbial communities in sponges (Hardoim et al., 2021). As in previous reports, these communities are composed mainly of unclassified taxa that need identification and description. A thorough description of the fauna associated with sponges is especially urgent as rapid environmental change may alter these communities unpredictably, leading to their disappearance.

## Conclusions

We characterized the prokaryotic and eukaryotic communities associated with *Tethya aurantium*, *Tethya meloni*, and *Tethya citrina*, three Mediterranean sponge species occurring in sympatry in the Mediterranean. We observed clear differences in microbiome composition between these sympatric sponge species, despite the high number of shared bacterial OTUs between species. Similarly, the eukaryotic communities associated with the three Mediterranean *Tethya* species showed clear differences between species. Our results provide evidence for a species-specific structuring of the prokaryotic and eukaryotic communities of sympatric congeneric sponges. These findings give insights into the ecology and evolution of sponge-associated communities.

## Supplemental Information

10.7717/peerj.20452/supp-1 Supplemental Information 1 Rarefaction curves for all 16S samples for each species separately with *Tethya aurantium* on the first row,* Tethya meloni* on the second row and *Tethya citrina* on the third

10.7717/peerj.20452/supp-2 Supplemental Information 2 Rarefaction curves for all 18S samples for each species separately with *Tethya aurantium* on the first row,* Tethya meloni* on the second row and *Tethya citrina* on the third

10.7717/peerj.20452/supp-3 Supplemental Information 3 High abundance 16S OTUs present at over 0.05 relative abundance in any individual sample (dots) compared across *Tethya* species in the three horizontal panels Phylum is indicated by colour which is secondarily placed under the OTU name and mean Relative abundance is denoted by the *y* axis position of the black central line on boxplots.

10.7717/peerj.20452/supp-4 Supplemental Information 4 Low abundance 16S core OTUs present under 0.05 relative abundance in any individual sample (dots) compared across *Tethya* species in the three horizontal panels Phylum is indicated by colour and mean Relative abundance is denoted by the *y* axis position of the black central line on boxplots.

10.7717/peerj.20452/supp-5 Supplemental Information 5 Deviance of different RAD models (left) and comparison of different RAD models(right) for the bacterial communities associated with three sympatric Mediterranean *Tethya* species

10.7717/peerj.20452/supp-6 Supplemental Information 6 NMDS ordination plots of Sorensen (presence-absence) 16S community dissimilarities across samples Mediterranean samples of *T. aurantium*, *T. meloni*, and *T. citrina* Each dot represents one sample. *Tethya meloni* samples are peach-colored, *T. aurantium* samples are colored purple-red, and *T. citrina* samples are blue.

10.7717/peerj.20452/supp-7 Supplemental Information 7 Heat plot showing the abundance of the top 25% most variable bacterial OTUs associated with three Mediterranean *Tethya* species The dendrogram was calculated using the default settings of the R function heatmap Samples belonging to *T. aurantium*, *T. meloni*, and *T. citrina* are labeled in Green, Red, and Blue, respectively, in the bar on the top.

10.7717/peerj.20452/supp-8 Supplemental Information 8 Heat plot showing the abundance of all bacterial OTUs associated with three Mediterranean *Tethya* species The dendrogram was calculated using the default settings of the R function heatmap Samples belonging to *T. aurantium*, *T. meloni*, and *T. citrina* are labeled in Green, Red, and Blue, respectively, in the bar on the top.

10.7717/peerj.20452/supp-9 Supplemental Information 9 High abundance 18S OTUs present under 0.05 relative abundance in any individual sample (dots) compared across *Tethya* species in the three horizontal panels Phylum is indicated by colour and mean Relative abundance is denoted by the *y* axis position of the black central line on boxplots.

10.7717/peerj.20452/supp-10 Supplemental Information 10 Deviance of different RAD models (left) and comparison of different RAD models (right) for the eukaryotic microbial communities associated with three sympatric Mediterranean *Tethya* species

10.7717/peerj.20452/supp-11 Supplemental Information 11 NMDS ordination plots of Sorensen (presence-absence) 18S community dissimilarities across samples Mediterranean samples of *T. aurantium*, *T. meloni*, and *T. citrina* Each dot represents one sample. *Tethya meloni* samples are peach-colored, *T. aurantium* samples are colored purple-red, and *T. citrina* samples are blue.

10.7717/peerj.20452/supp-12 Supplemental Information 12 Heat plot showing the abundance of the top 25% most variable eukaryotic microbial OTUs associated with three Mediterranean *Tethya* species The dendrogram was calculated using the default settings of the R function heatmap Samples belonging to *T. aurantium*, *T. meloni*, and *T. citrina* are labeled in Green, Red, and Blue, respectively, in the bar on the top.

10.7717/peerj.20452/supp-13 Supplemental Information 13 Supplementary tables

10.7717/peerj.20452/supp-14 Supplemental Information 14 Figure legends and supplementary figure legends

## Additional Information and Declarations

### Data Availability

The following information was supplied regarding data availability: The data is available at ENA under Bioproject PRJEB91742. The code is available at Github and Zenodo: – https://github.com/PalMuc/MediterraneanTethyaMicrobiomes
– Sergio Vargas. (2025). PalMuc/MediterraneanTethyaMicrobiomes: 20251112_Zenodo_Sync (zenodo). Zenodo. https://doi.org/10.5281/zenodo.17594208.
