# Supplementary figures and images for: Species-specific community structure in the microbiomes and eukaryotic communities associated with Mediterranean golf ball sponges
Source: PeerJ. 2026 Mar 10;14:e20452. doi: 10.7717/peerj.20452 (PMC12985019; doi:10.7717/peerj.20452)

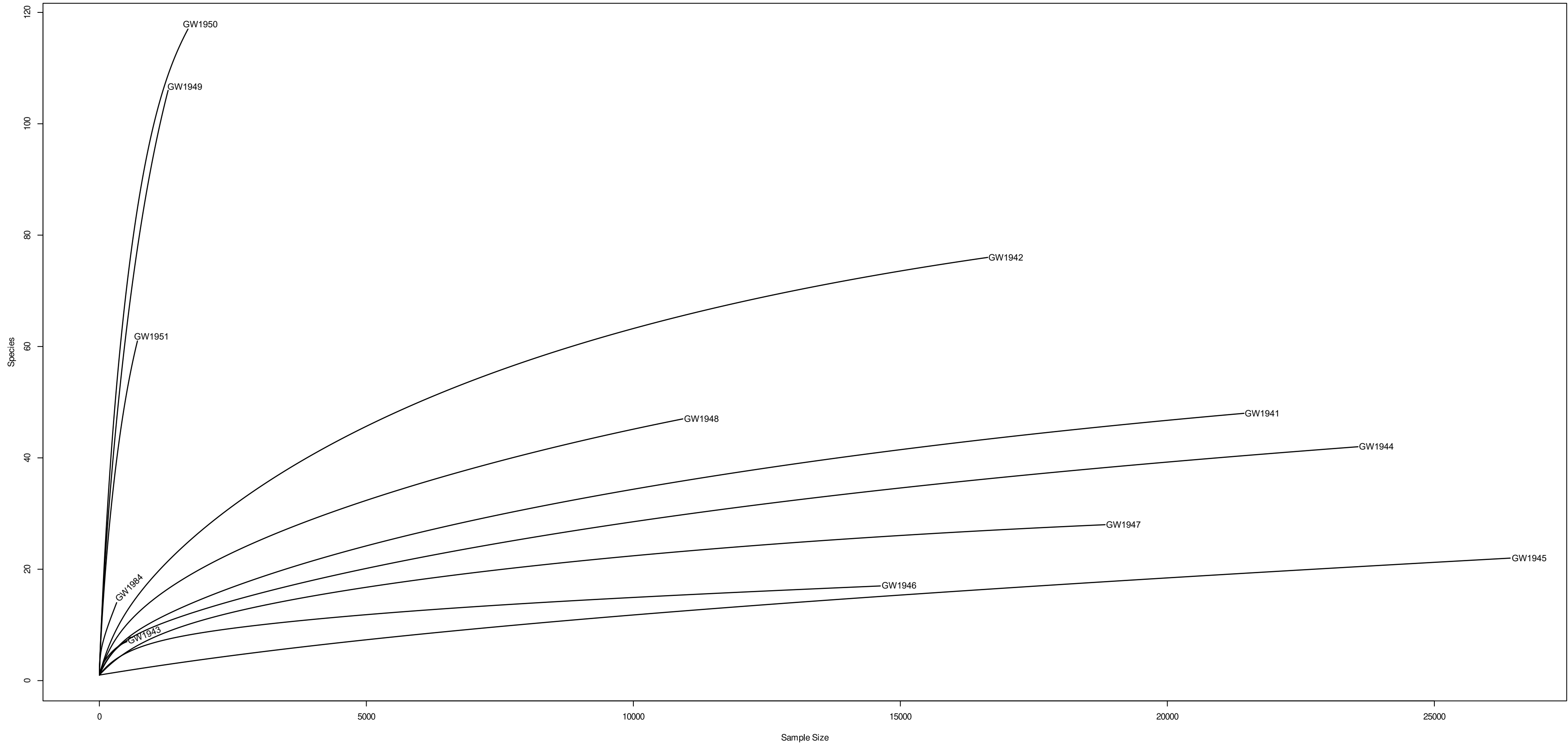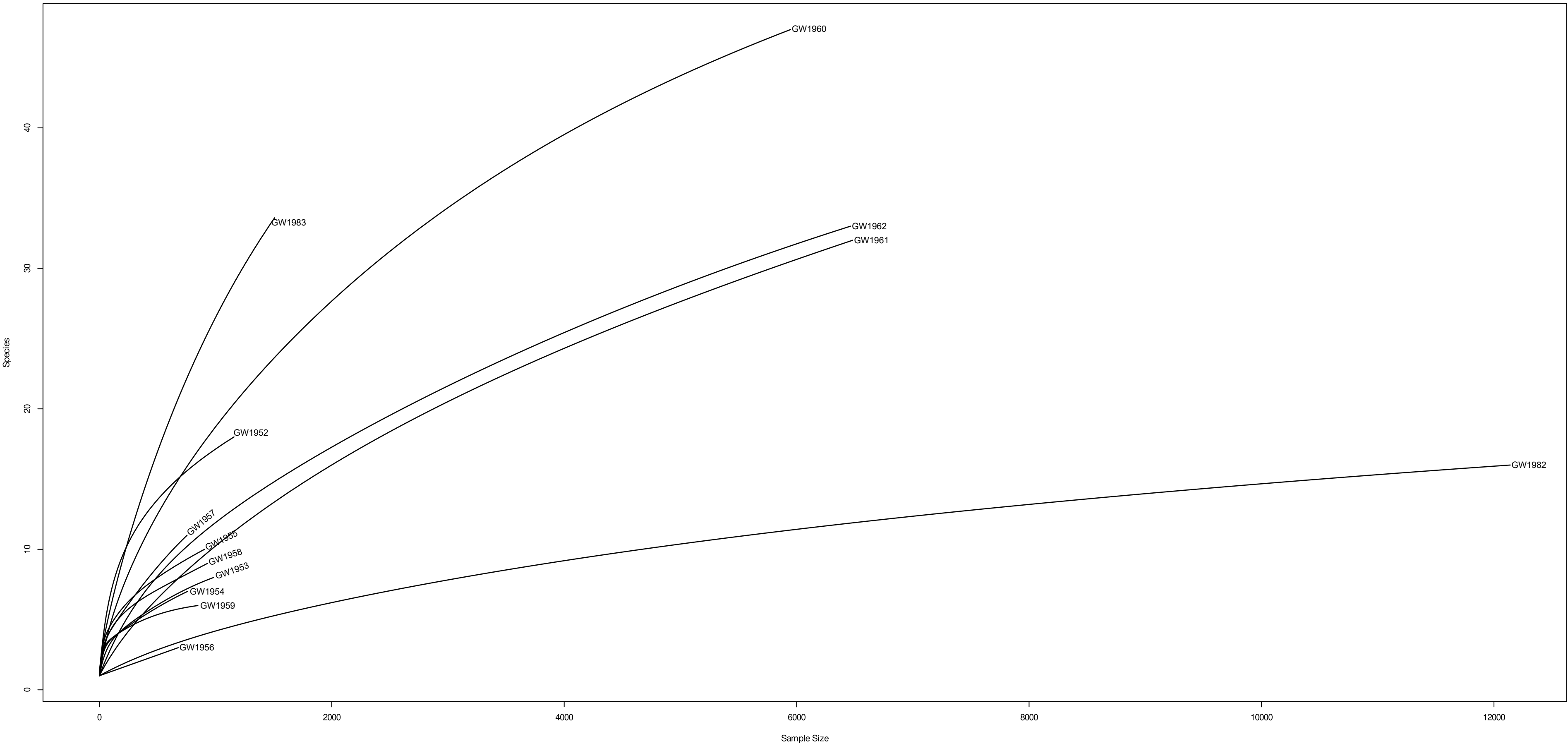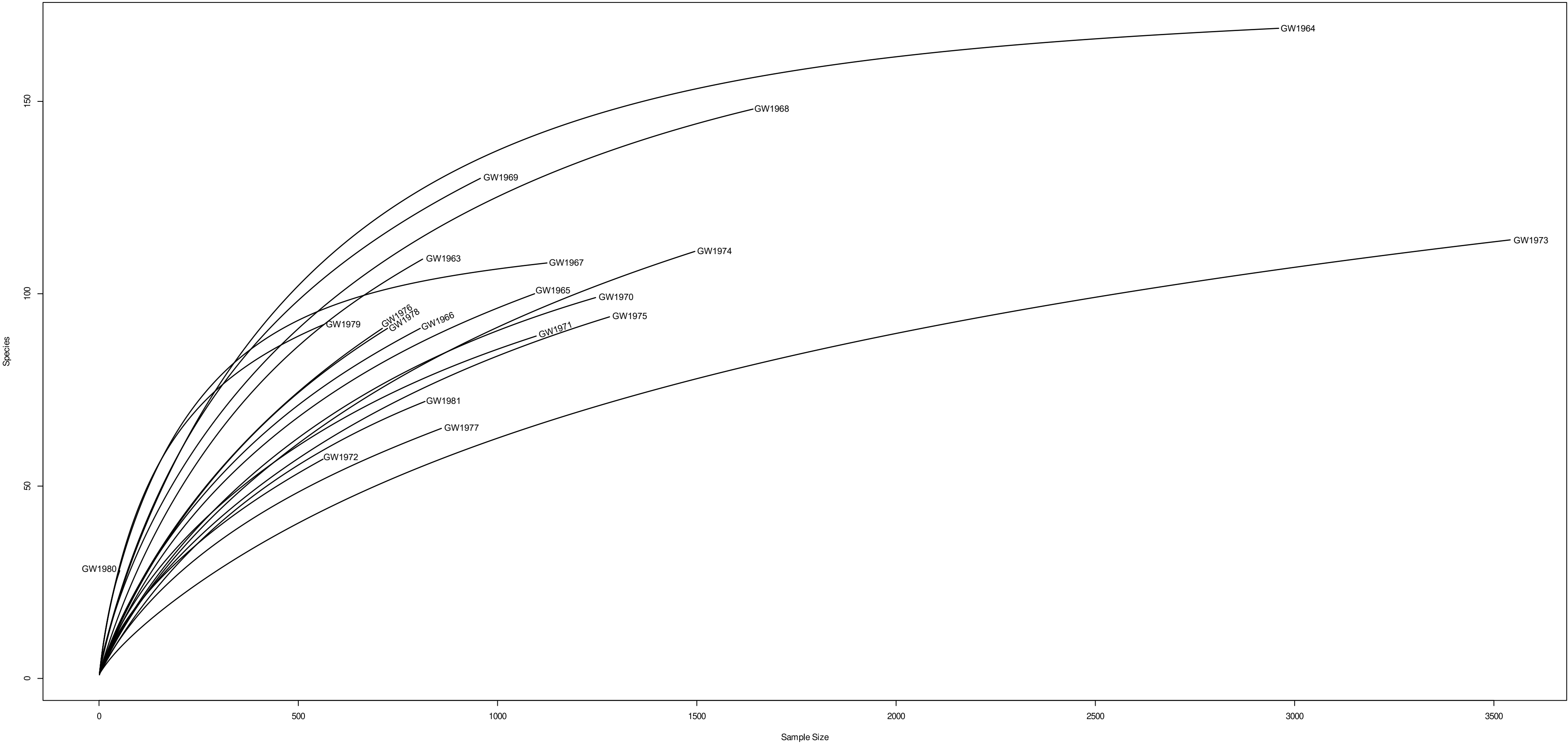

Supplement: Supplemental Information 1 [file peerj-14-20452-s001.pdf]

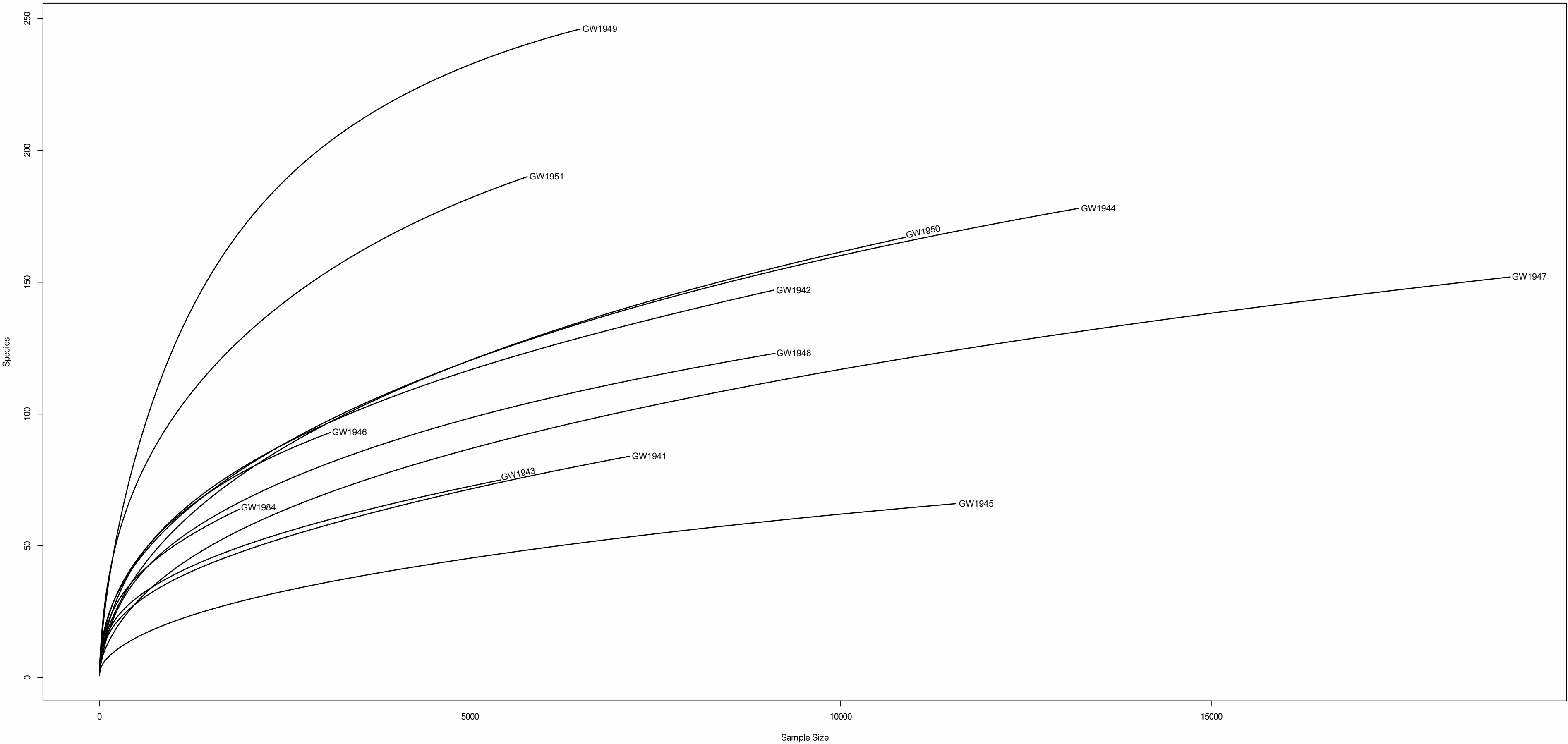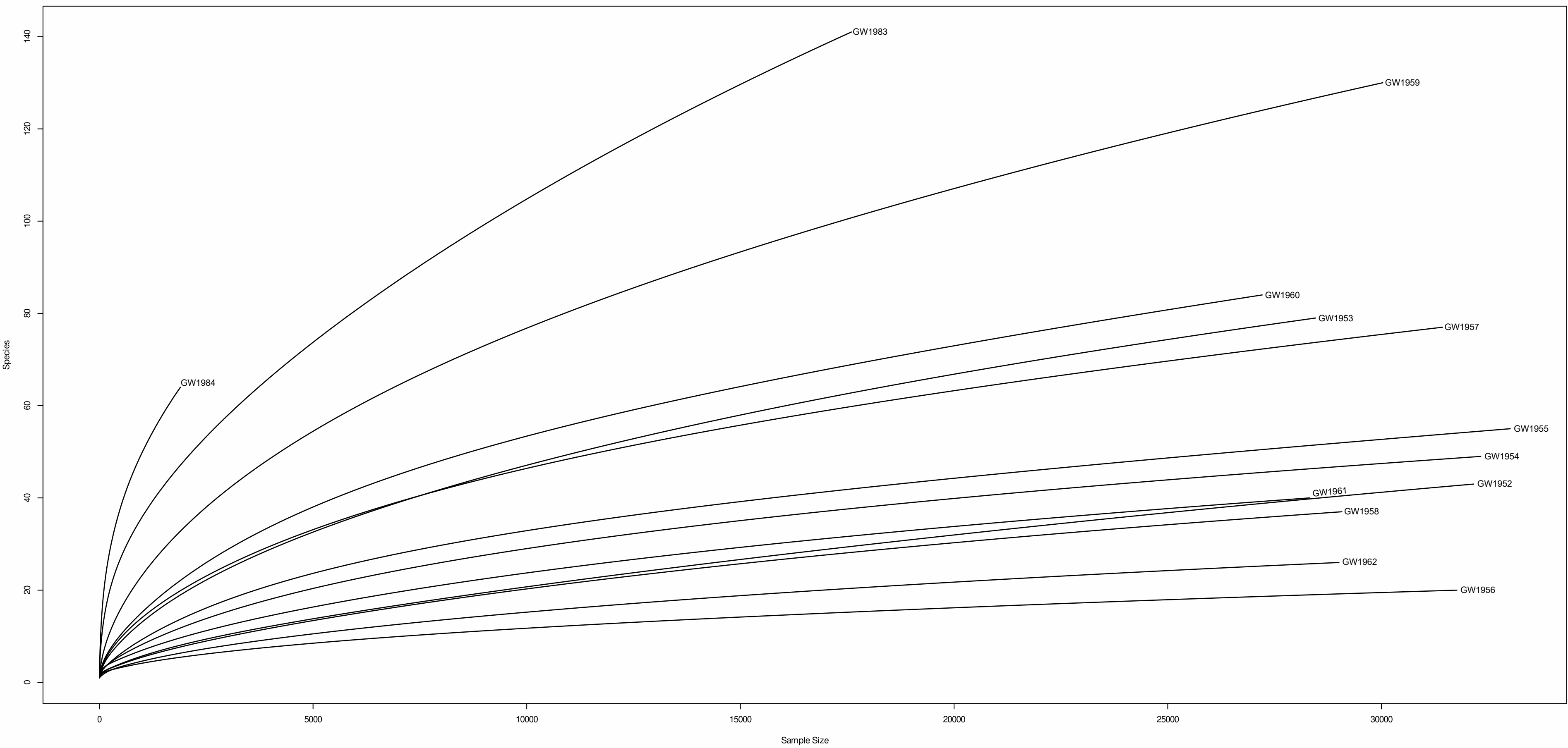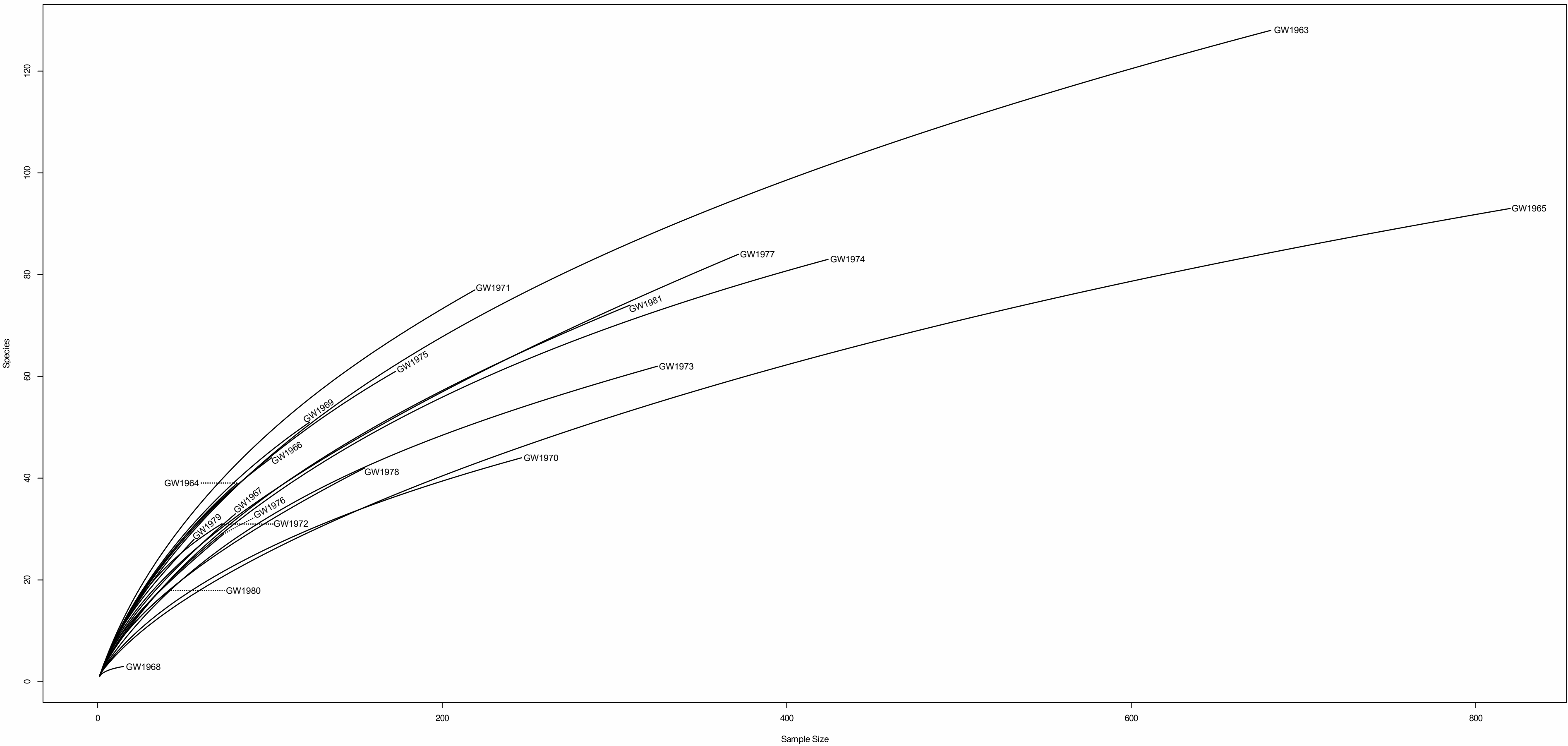

Supplement: Supplemental Information 2 [file peerj-14-20452-s002.pdf]

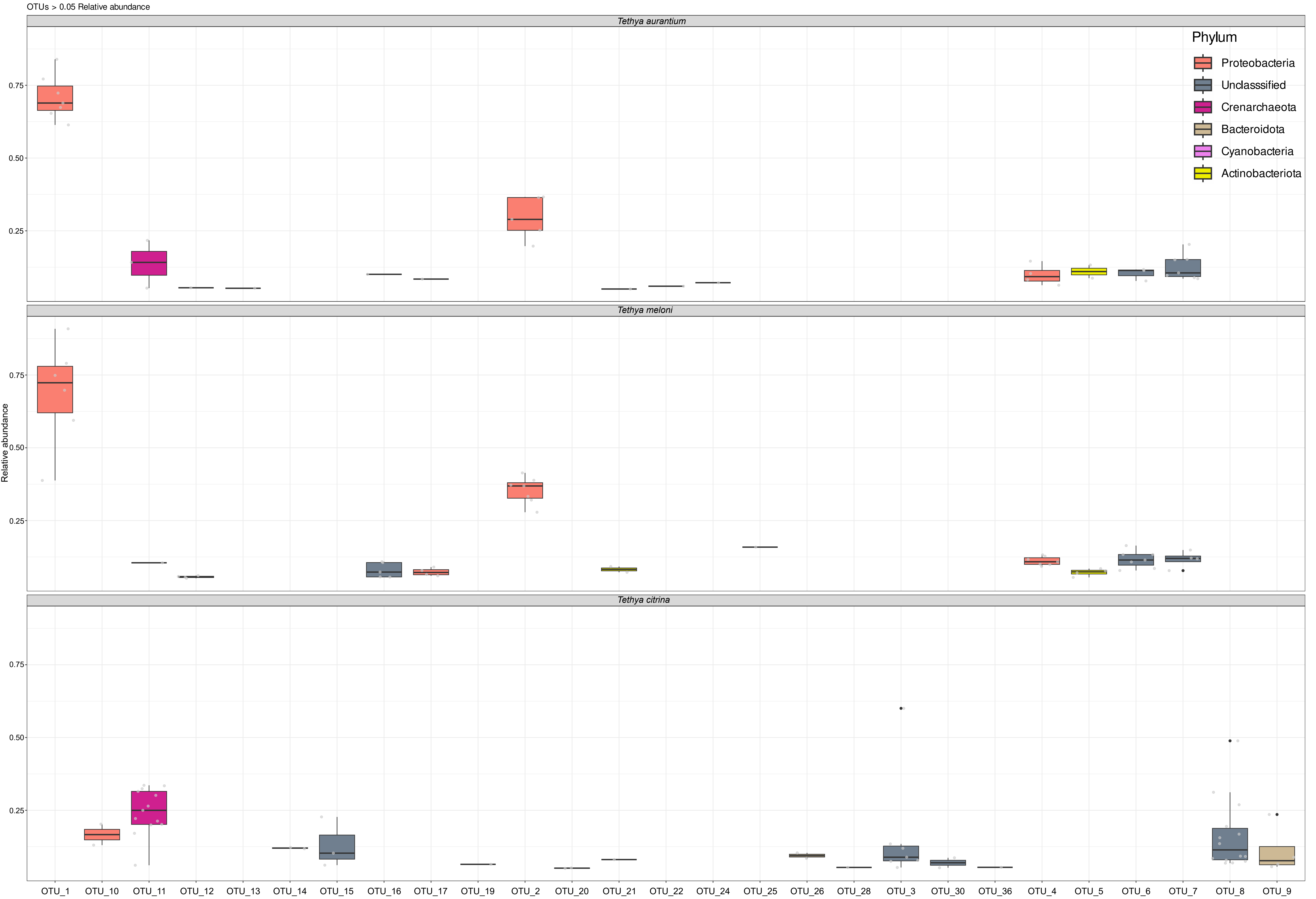

Supplement: Supplemental Information 3 — Phylum is indicated by colour which is secondarily placed under the OTU name and mean Relative abundance is denoted by the y axis position of the black central line on boxplots. [file peerj-14-20452-s003.pdf]

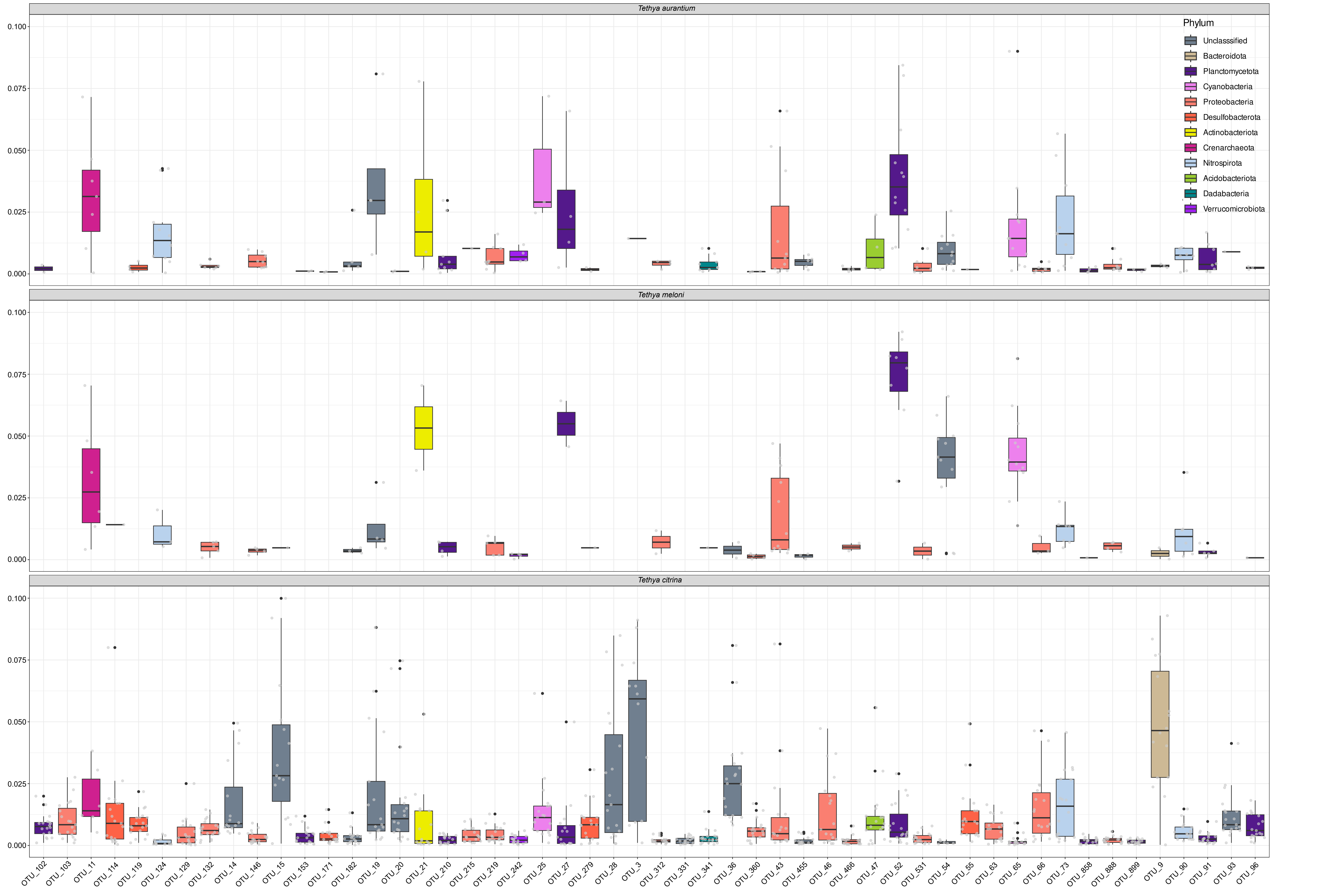

Supplement: Supplemental Information 4 — Phylum is indicated by colour and mean Relative abundance is denoted by the y axis position of the black central line on boxplots. [file peerj-14-20452-s004.pdf]

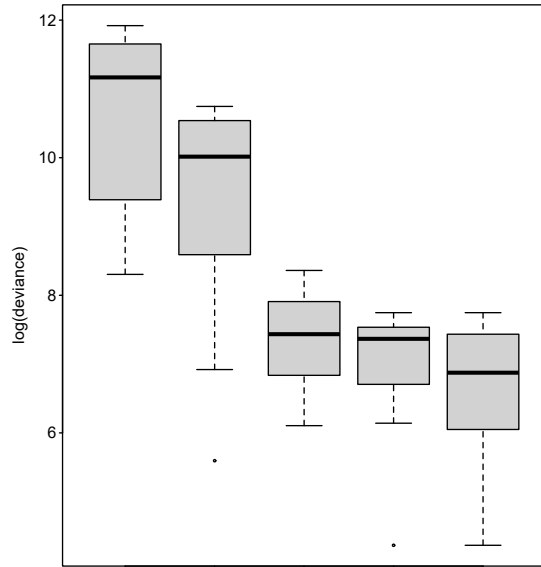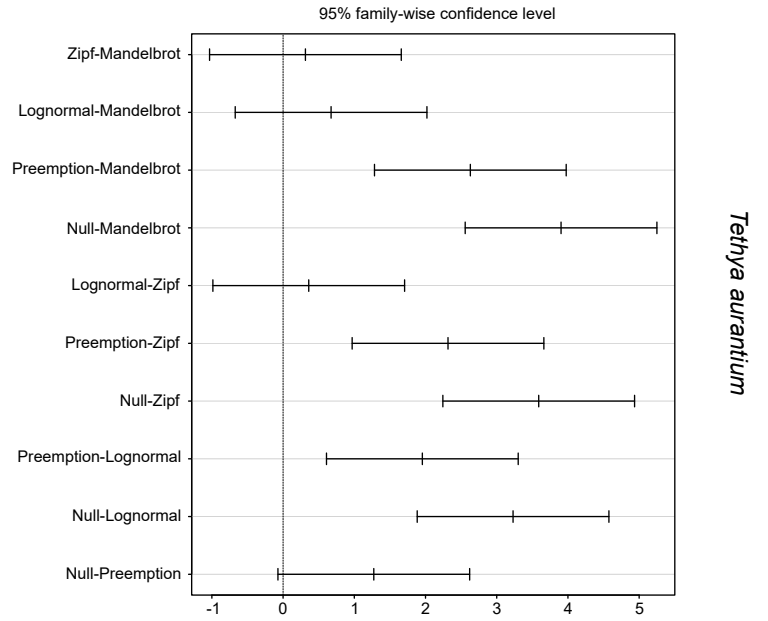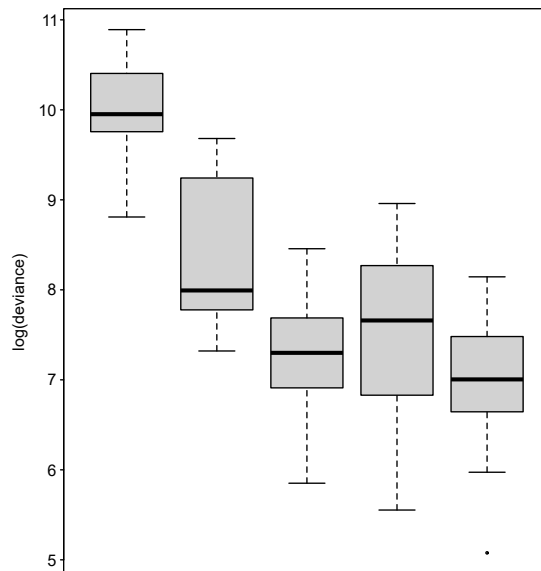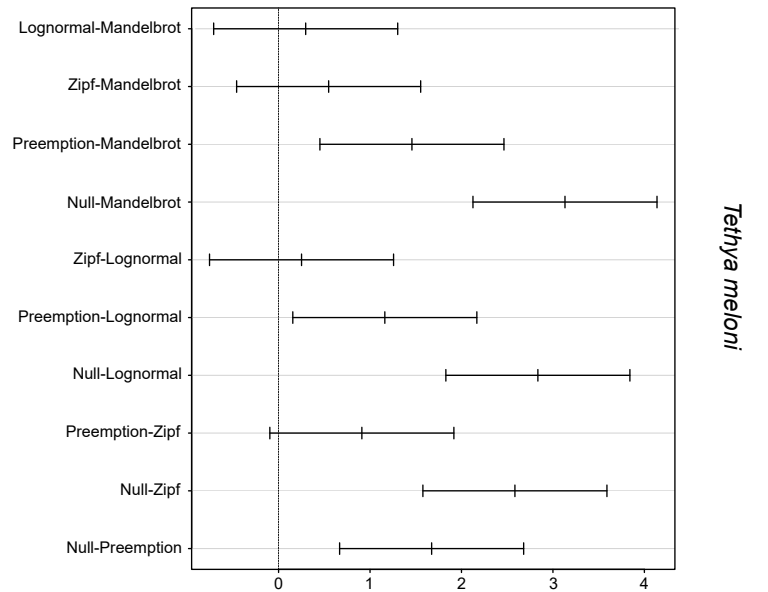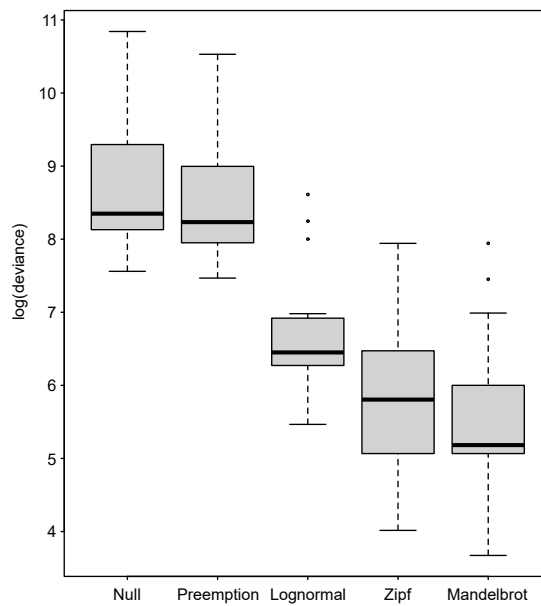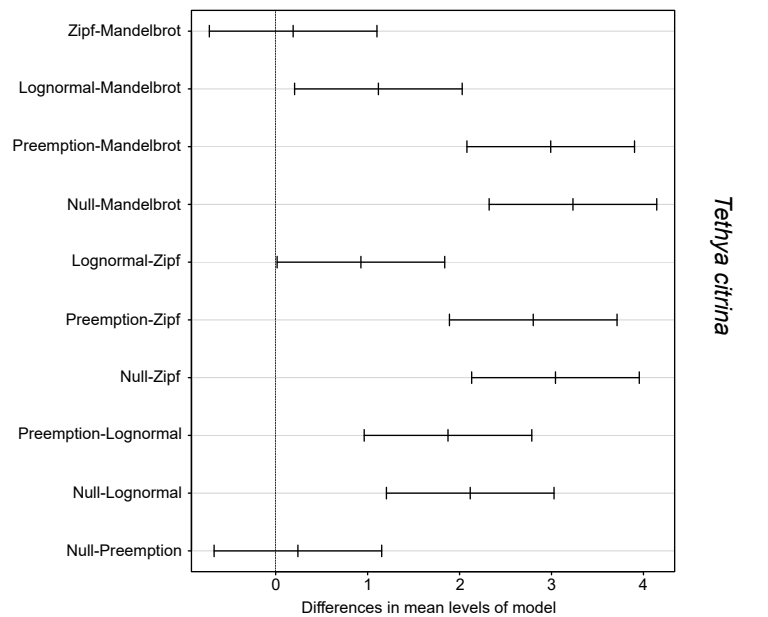

Supplement: Supplemental Information 5 [file peerj-14-20452-s005.pdf]

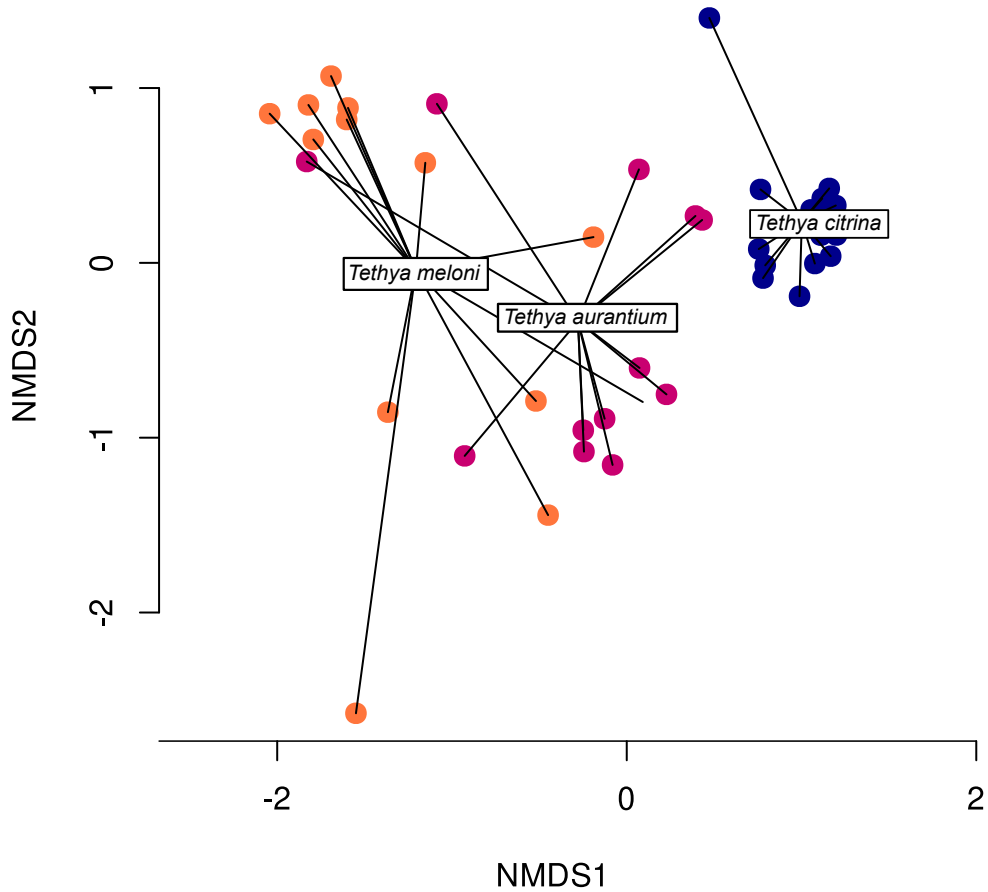

Supplement: Supplemental Information 6 — Each dot represents one sample. Tethya meloni samples are peach-colored, T. aurantium samples are colored purple-red, and T. citrina samples are blue. [file peerj-14-20452-s006.pdf]

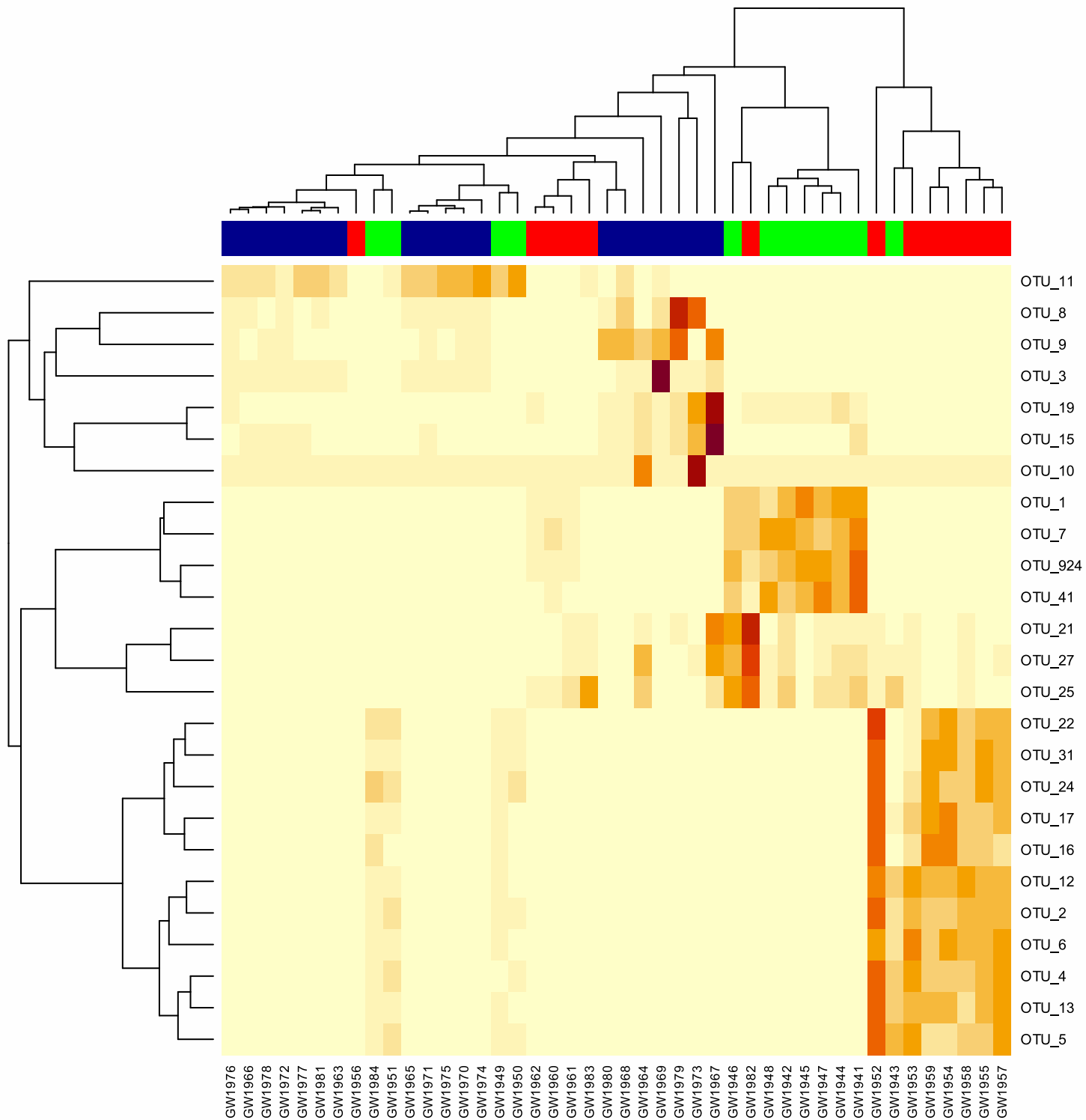

Supplement: Supplemental Information 7 — The dendrogram was calculated using the default settings of the R function heatmap Samples belonging to T. aurantium, T. meloni, and T. citrina are labeled in Green, Red, and Blue, respectively, in the bar on the top. [file peerj-14-20452-s007.pdf]

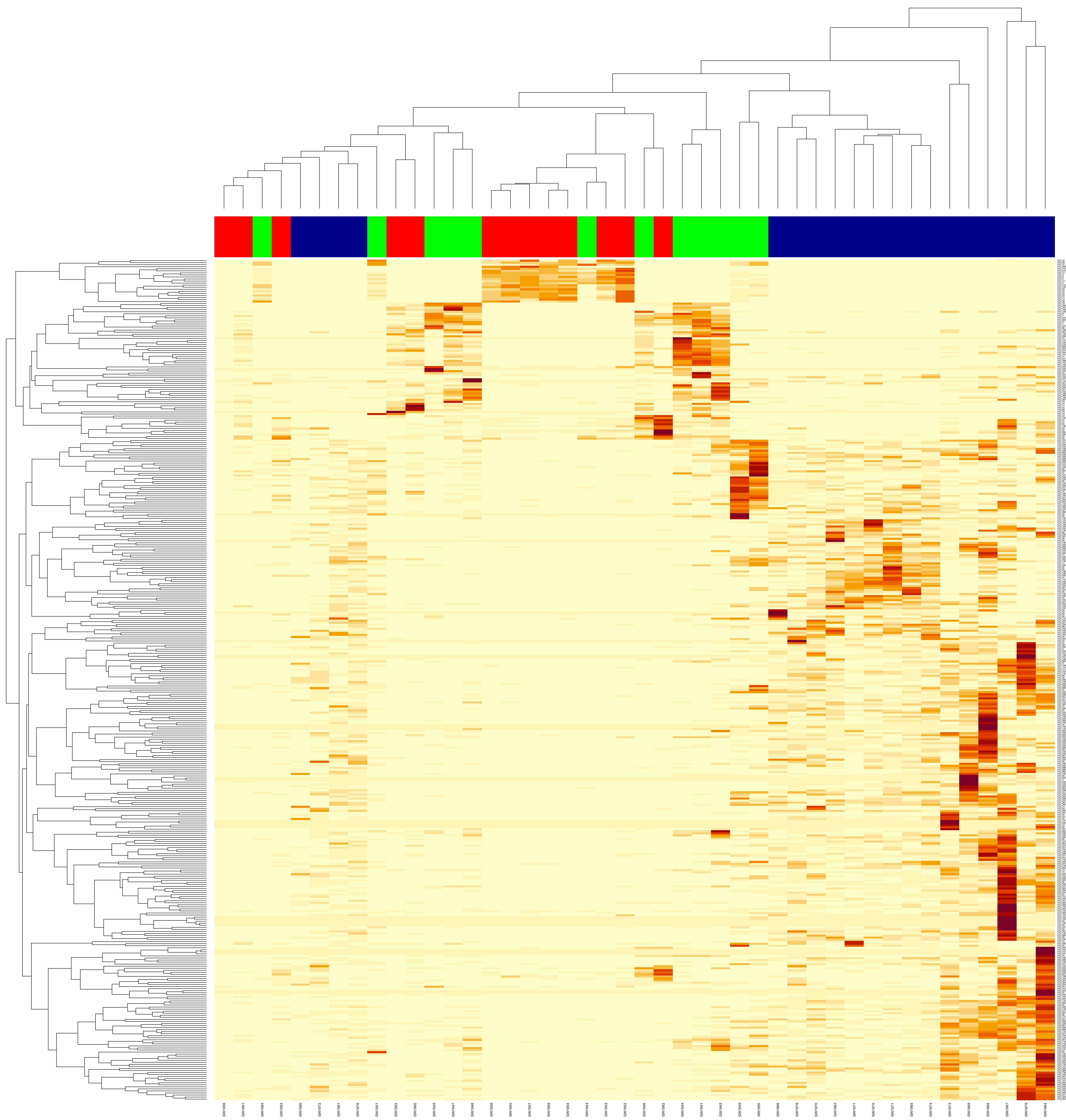

Supplement: Supplemental Information 8 — The dendrogram was calculated using the default settings of the R function heatmap Samples belonging to T. aurantium, T. meloni, and T. citrina are labeled in Green, Red, and Blue, respectively, in the bar on the top. [file peerj-14-20452-s008.pdf]

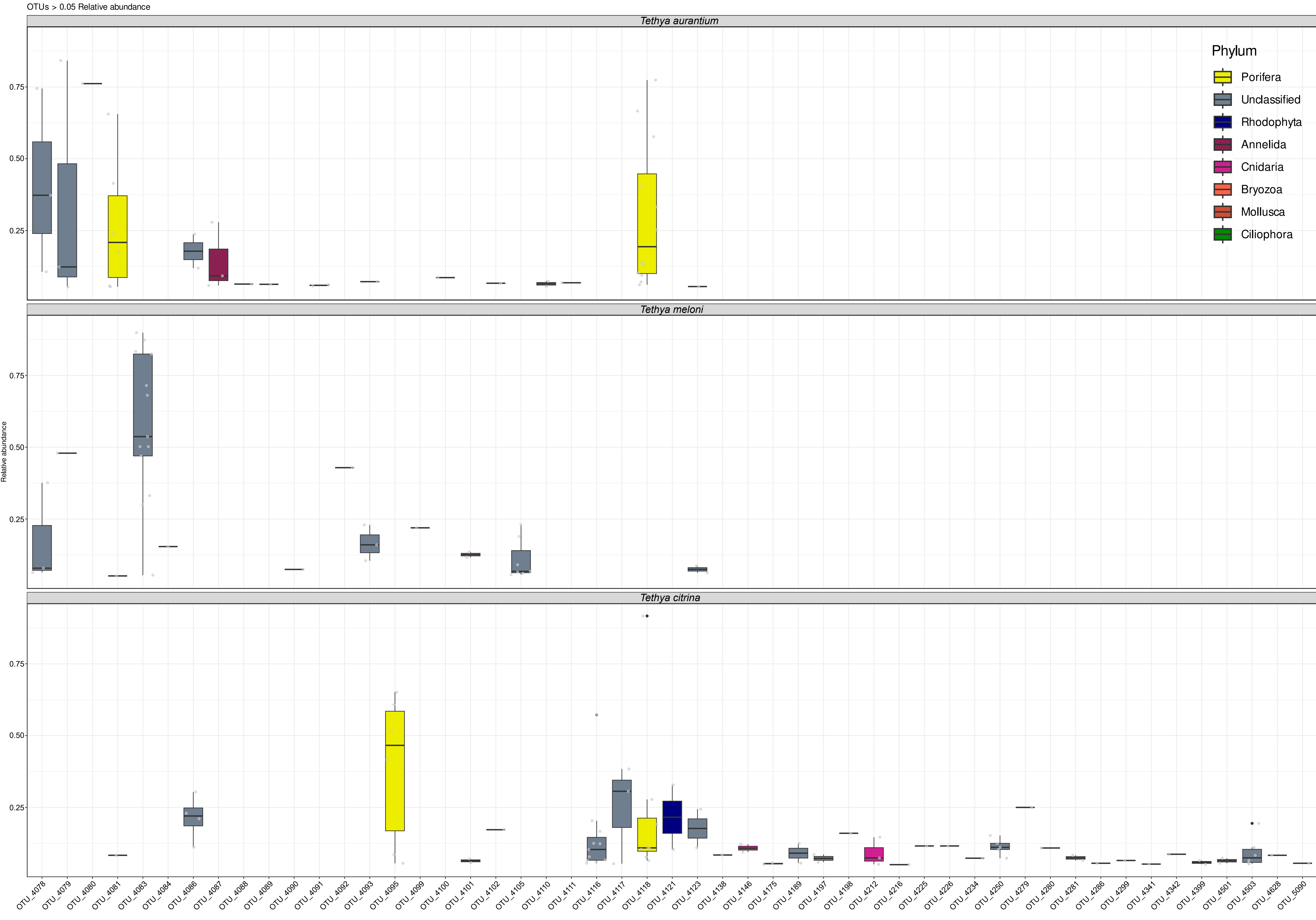

Supplement: Supplemental Information 9 — Phylum is indicated by colour and mean Relative abundance is denoted by the y axis position of the black central line on boxplots. [file peerj-14-20452-s009.pdf]

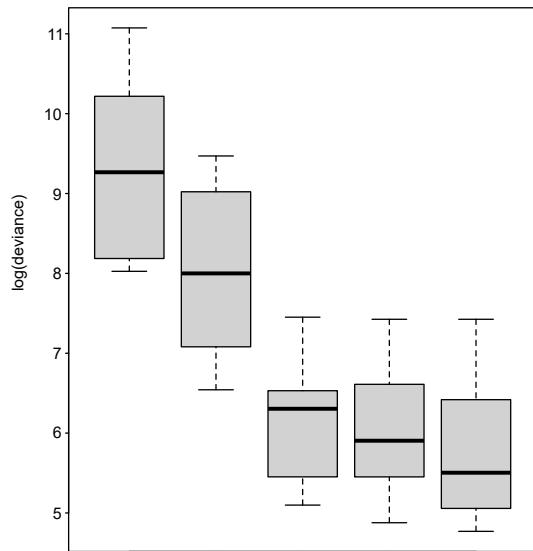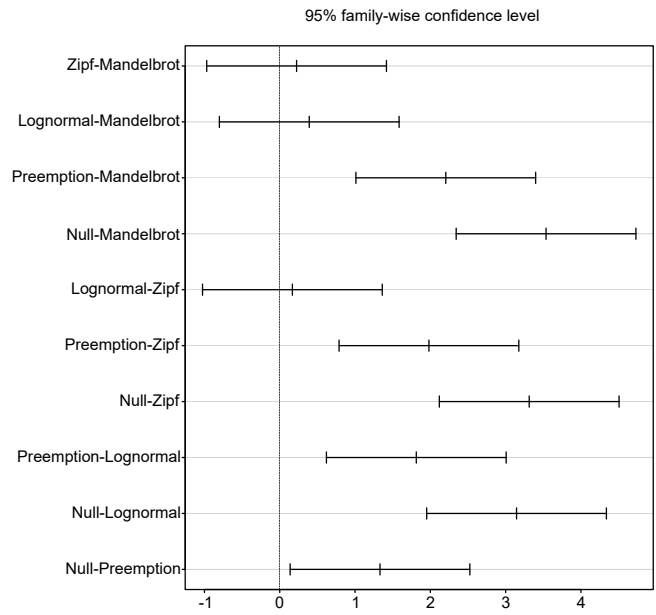

*Tethya aurantium*

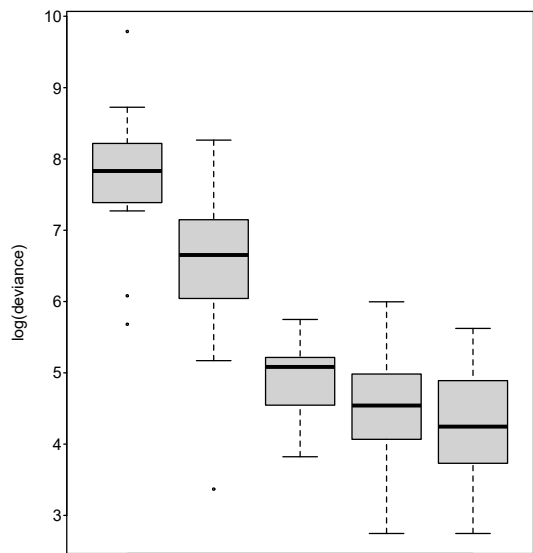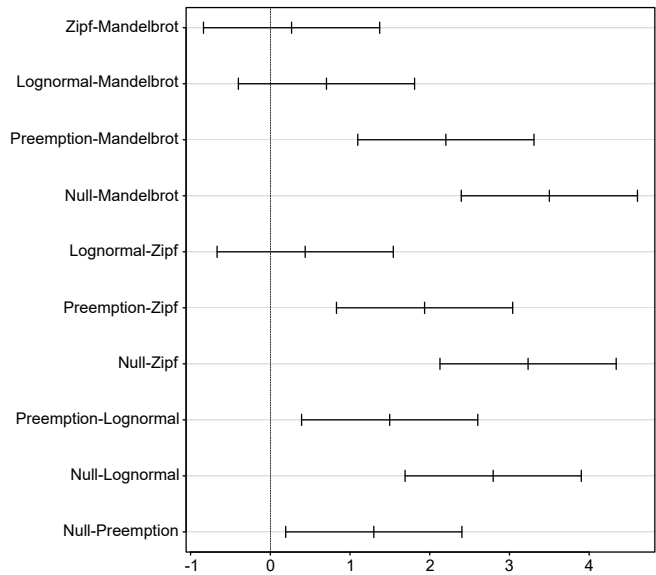

*Tethya meloni*

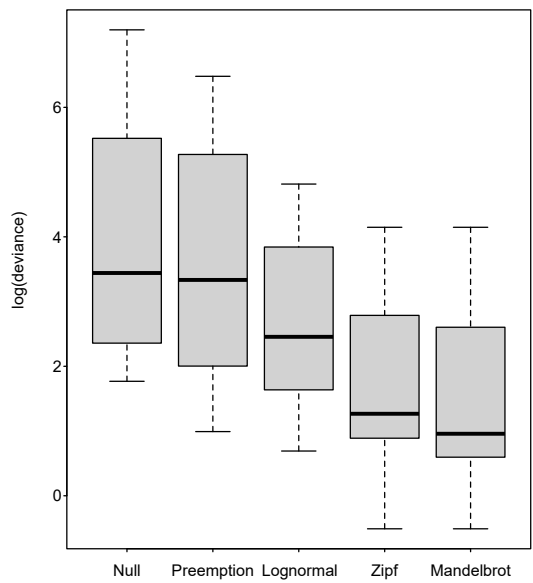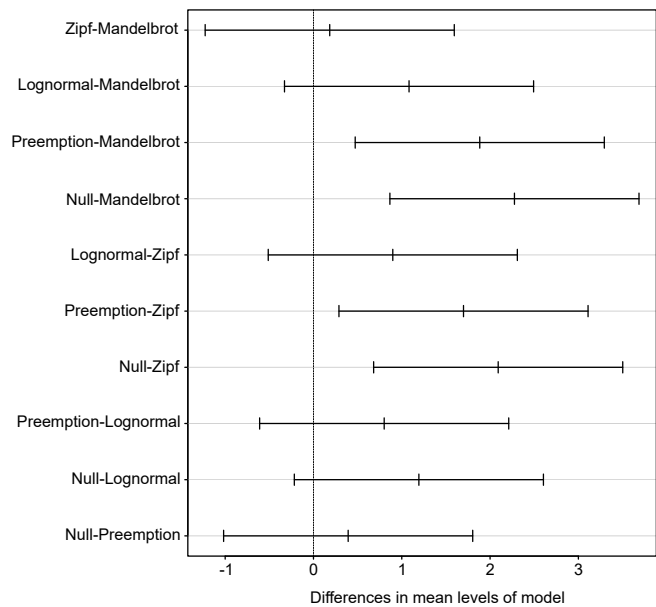

*Tethya citrina*

Differences in mean levels of model

Supplement: Supplemental Information 10 [file peerj-14-20452-s010.pdf]

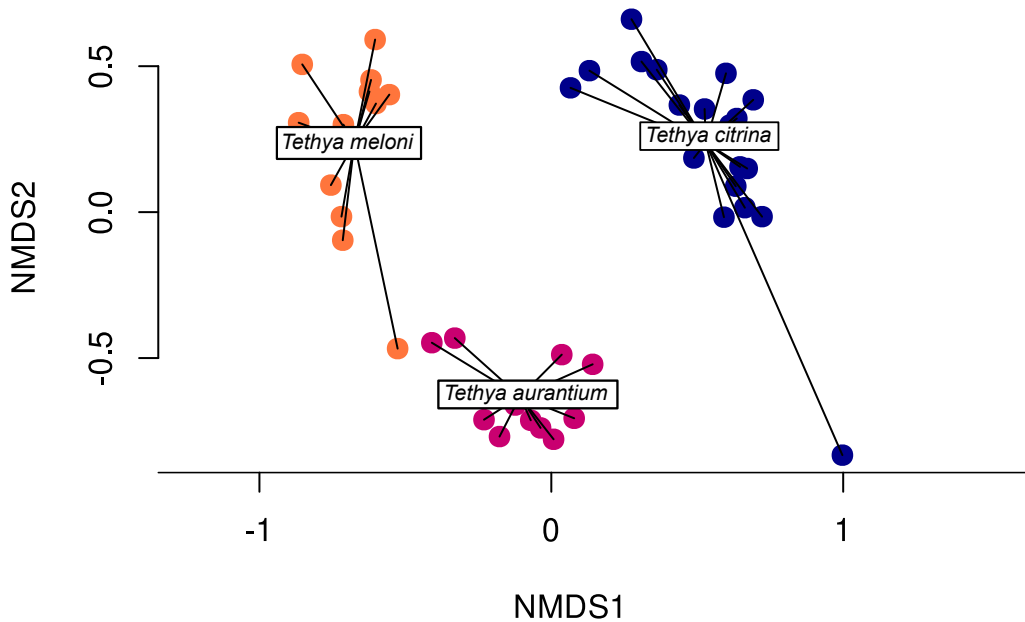

Supplement: Supplemental Information 11 — Each dot represents one sample. Tethya meloni samples are peach-colored, T. aurantium samples are colored purple-red, and T. citrina samples are blue. [file peerj-14-20452-s011.pdf]

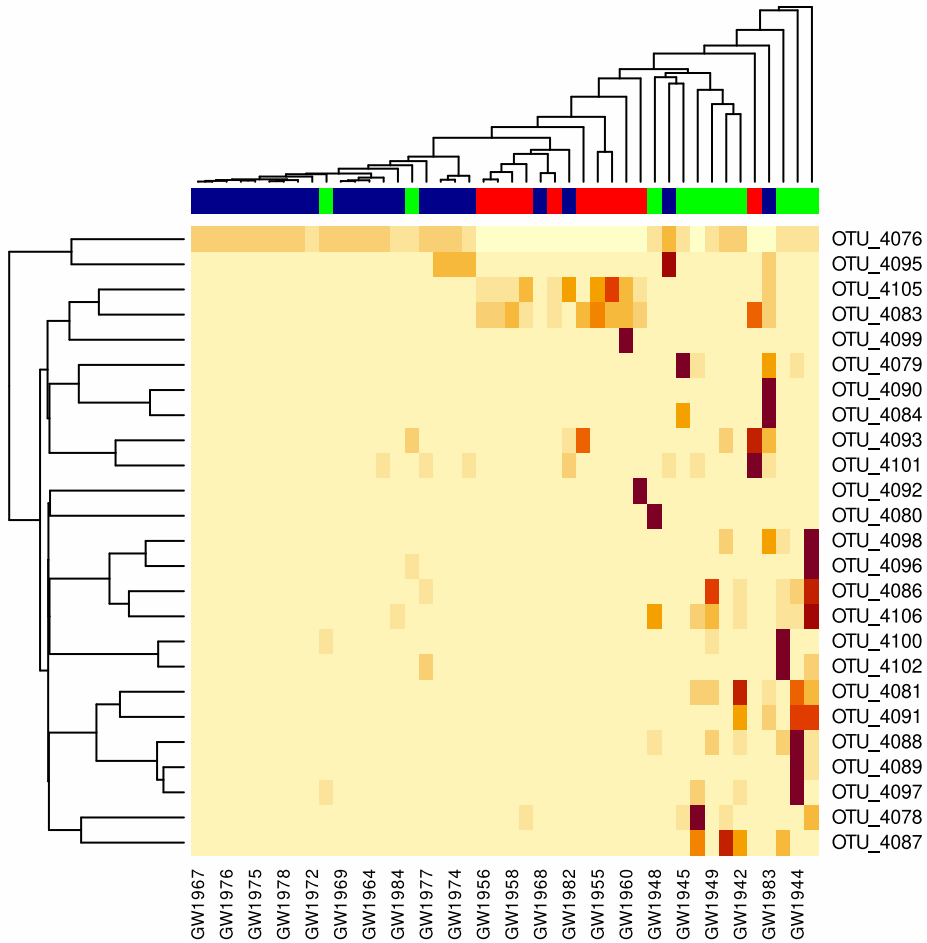

Supplement: Supplemental Information 12 — The dendrogram was calculated using the default settings of the R function heatmap Samples belonging to T. aurantium, T. meloni, and T. citrina are labeled in Green, Red, and Blue, respectively, in the bar on the top. [file peerj-14-20452-s012.pdf]
