# Supplementary material for: Species-specific community structure in the microbiomes and eukaryotic communities associated with Mediterranean golf ball sponges
Source: PeerJ. 2026 Mar 10;14:e20452. doi: 10.7717/peerj.20452 (PMC12985019; doi:10.7717/peerj.20452)
Supplement: Supplemental Information 13 [file peerj-14-20452-s013.docx]

Supplementary Table 1. Universal 18S rRNA primers Euk1391f and EukBr adapted following Pichler et al. XXXX. The primers consist of the Illumina P5/P7 adaptor (bold), and index (underlined), a pad (italics), and a linker (double underline), and have the EukBr or Euk1391f primer at the 3’ end.

| Barcode name | Barcode sequence | Final Primer | Primer Name |
| --- | --- | --- | --- |
| N701 | TCGCCTTA | **CAAGCAGAAGACGGCATACGAGAT**TCGCCTTA*AGTCAGTCAG*CCTGATCCTTCTGCAGGTTCACCTAC | N701_18S-EukBr |
| N702 | CTAGTACG | CAAGCAGAAGACGGCATACGAGATCTAGTACGAGTCAGTCAGCCTGATCCTTCTGCAGGTTCACCTAC | N702_18S-EukBr |
| N703 | TTCTGCCT | CAAGCAGAAGACGGCATACGAGATTTCTGCCTAGTCAGTCAGCCTGATCCTTCTGCAGGTTCACCTAC | N703_18S-EukBr |
| N704 | GCTCAGGA | CAAGCAGAAGACGGCATACGAGATGCTCAGGAAGTCAGTCAGCCTGATCCTTCTGCAGGTTCACCTAC | N704_18S-EukBr |
| N705 | AGGAGTCC | CAAGCAGAAGACGGCATACGAGATAGGAGTCCAGTCAGTCAGCCTGATCCTTCTGCAGGTTCACCTAC | N705_18S-EukBr |
| N706 | CATGCCTA | CAAGCAGAAGACGGCATACGAGATCATGCCTAAGTCAGTCAGCCTGATCCTTCTGCAGGTTCACCTAC | N706_18S-EukBr |
| N707 | GTAGAGAG | CAAGCAGAAGACGGCATACGAGATGTAGAGAGAGTCAGTCAGCCTGATCCTTCTGCAGGTTCACCTAC | N707_18S-EukBr |
| N708 | CCTCTCTG | CAAGCAGAAGACGGCATACGAGATCCTCTCTGAGTCAGTCAGCCTGATCCTTCTGCAGGTTCACCTAC | N708_18S-EukBr |
| N709 | AGCGTAGC | CAAGCAGAAGACGGCATACGAGATAGCGTAGCAGTCAGTCAGCCTGATCCTTCTGCAGGTTCACCTAC | N709_18S-EukBr |
| N710 | CAGCCTCG | CAAGCAGAAGACGGCATACGAGATCAGCCTCGAGTCAGTCAGCCTGATCCTTCTGCAGGTTCACCTAC | N710_18S-EukBr |
| N711 | TGCCTCTT | CAAGCAGAAGACGGCATACGAGATTGCCTCTTAGTCAGTCAGCCTGATCCTTCTGCAGGTTCACCTAC | N711_18S-EukBr |
| N712 | TCCTCTAC | CAAGCAGAAGACGGCATACGAGATTCCTCTACAGTCAGTCAGCCTGATCCTTCTGCAGGTTCACCTAC | N712_18S-EukBr |
| N502 | CTCTCTAT | **AATGATACGGCGACCACCGAGATCTACAC**CTCTCTAT*TATGGTAATT*GTGTACACACCGCCCGTC | N502_18S-1391F |
| N503 | TATCCTCT | AATGATACGGCGACCACCGAGATCTACACTATCCTCTTATGGTAATTGTGTACACACCGCCCGTC | N503_18S-1391F |
| N504 | AGAGTAGA | AATGATACGGCGACCACCGAGATCTACACAGAGTAGATATGGTAATTGTGTACACACCGCCCGTC | N504_18S-1391F |
| N505 | GTAAGGAG | AATGATACGGCGACCACCGAGATCTACACGTAAGGAGTATGGTAATTGTGTACACACCGCCCGTC | N505_18S-1391F |
| N506 | ACTGCATA | AATGATACGGCGACCACCGAGATCTACACACTGCATATATGGTAATTGTGTACACACCGCCCGTC | N506_18S-1391F |
| N507 | AAGGAGTA | AATGATACGGCGACCACCGAGATCTACACAAGGAGTATATGGTAATTGTGTACACACCGCCCGTC | N507_18S-1391F |
| N508 | CTAAGCCT | AATGATACGGCGACCACCGAGATCTACACCTAAGCCTTATGGTAATTGTGTACACACCGCCCGTC | N508_18S-1391F |

Supplementary Table 2. Canonical correspondence analyses of the bacterial communities associated with three sympatric Mediterranean *Tethya* species. We tested for differences between species using 999 permutations.

|  | DF | ChiSquare | F | Pr(>F) |
| --- | --- | --- | --- | --- |
| Model | 2 | 1.2388 | 9.3219 | 0.001 |
| Residual | 41 | 2.7242 |  |  |
