# Supplementary material for: Species-specific community structure in the microbiomes and eukaryotic communities associated with Mediterranean golf ball sponges
Source: PeerJ. 2026 Mar 10;14:e20452. doi: 10.7717/peerj.20452 (PMC12985019; doi:10.7717/peerj.20452)
Supplement: Supplemental Information 14 [file peerj-14-20452-s014.docx]

Figure Legends

Figure 1. Map of sampling sites for *T. aurantium*, *T. meloni* and *T. citrina* at Mar Piccolo. Colour indicates depth of the lagoon and the grey dots represent the sampling location for each species.

Figure 2. 16S OTUs richness across phyla present for all species (left) and the three *Tethya* species separately (right). The color of the bars in all plots is indicative of the taxonomic group.

Figure 3. 16S OTU overlap between species of Mediterranean *Tethya.* Left: whole microbiome. Right: core 16S OTUs (i.e., OTUs present in ≥90% of a species’ samples).

Figure 4. Phylum OTU richness and relative abundance in the whole and core microbiome of Mediterranean *Tethya* species.

Figure 5. Abundance-rank dominance models fitted to OTU data for the 16S dataset. For each species (a. *T. aurantium,* b. *T. meloni,* c. *T. citrina*), the sample replicates (represented as lines) are drawn under the best-fit model for the distribution of the OTU count data (represented as circles) and grouped into the same box under the name of the assigned model.

Figure 6. NMDS ordination plots of Bray-Curtis 16S community dissimilarities across samples Mediterranean samples of *T. aurantium*, *T. meloni*, and *T. citrina*. Each dot represents one sample. *Tethya meloni* samples are peach-colored, *T. aurantium* samples are colored purple-red, and *T. citrina* samples are blue.

Figure 7. 18S OTUs richness across phyla present for all species (top) and the three *Tethya* species separately (bottom). The color of the bars in all plots is indicative of the taxonomic group.

Figure 8. 18S OTU overlap between species of Mediterranean *Tethya.* Left: core community (OTUs present in 90% of samples). Right: all 18S OTUs.

Figure 9. Relative abundance of 18S OTUs grouped into phyla for each *Tethya* species for (a) all OTUs and (b) excluding unclassified OTUs and OTUs belonging to Phylum Porifera.

Figure 10. Rank-abundance dominance models fitted to OTU data for the 18S dataset. For each species (i.e., *T. aurantium, T. meloni,* and *T. citrina*), the replicates (represented as lines) are drawn under the best-fit model for the distribution of the OTU count data (represented as circles) and grouped into the same box under the name of the assigned model.

Figure 11. NMDS ordination plots of Bray-Curtis distances for 18S community structure across samples grouped by species. Each dot represents one sample. *Tethya meloni* samples are peach-colored, *T. aurantium* samples are colored purple-red, and *T. citrina* samples are blue.

Supplementary Figure Legends

Figure S1. Rarefaction curves for all 16S samples for each species separately with *Tethya aurantium* on the first row, *Tethya meloni* on the second row and *Tethya citrina* on the third.

Figure S2. Rarefaction curves for all 18S samples for each species separately with *Tethya aurantium* on the first row, *Tethya meloni* on the second row and *Tethya citrina* on the third.

Figure S3. High abundance 16S OTUs present at over 0.05 relative abundance in any individual sample (dots) compared across *Tethya* species in the three horizontal panels. Phylum is indicated by colour which is secondarily placed under the OTU name and mean Relative abundance is denoted by the y axis position of the black central line on boxplots.

Figure S4. Low abundance 16S core OTUs present under 0.05 relative abundance in any individual sample (dots) compared across *Tethya* species in the three horizontal panels. Phylum is indicated by colour and mean Relative abundance is denoted by the y axis position of the black central line on boxplots.

Figure S5. Deviance of different RAD models (left) and comparison of different RAD models(right) for the bacterial communities associated with three sympatric Mediterranean *Tethya* species.

Figure S6. NMDS ordination plots of Sorensen (presence-absence) 16S community dissimilarities across samples Mediterranean samples of *T. aurantium*, *T. meloni*, and *T. citrina*. Each dot represents one sample. *Tethya meloni* samples are peach-colored, *T. aurantium* samples are colored purple-red, and *T. citrina* samples are blue.

Figure S7. Heat plot showing the abundance of the top 25% most variable bacterial OTUs associated with three Mediterranean *Tethya* species. The dendrogram was calculated using the default settings of the R function heatmap(). Samples belonging to *T. aurantium*, *T. meloni*, and *T. citrina* are labeled in Green, Red, and Blue, respectively, in the bar on the top.

Figure S8. Heat plot showing the abundance of all bacterial OTUs associated with three Mediterranean *Tethya* species. The dendrogram was calculated using the default settings of the R function heatmap(). Samples belonging to *T. aurantium*, *T. meloni*, and *T. citrina* are labeled in Green, Red, and Blue, respectively, in the bar on the top.

Figure S9. High abundance 18S OTUs present under 0.05 relative abundance in any individual sample (dots) compared across *Tethya* species in the three horizontal panels. Phylum is indicated by colour and mean Relative abundance is denoted by the y axis position of the black central line on boxplots.

Figure S10. Deviance of different RAD models (left) and comparison of different RAD models(right) for the eukaryotic microbial communities associated with three sympatric Mediterranean *Tethya* species.

Figure S11. NMDS ordination plots of Sorensen (presence-absence) 18S community dissimilarities across samples Mediterranean samples of *T. aurantium*, *T. meloni*, and *T. citrina*. Each dot represents one sample. *Tethya meloni* samples are peach-colored, *T. aurantium* samples are colored purple-red, and *T. citrina* samples are blue.

Figure S12. Heat plot showing the abundance of the top 25% most variable eukaryotic microbial OTUs associated with three Mediterranean *Tethya* species. The dendrogram was calculated using the default settings of the R function heatmap(). (). Samples belonging to *T. aurantium*, *T. meloni*, and *T. citrina* are labeled in Green, Red, and Blue, respectively, in the bar on the top.
